# Altered protein turnover signaling and myogenesis during impaired recovery of inflammation-induced muscle atrophy in emphysematous mice

**DOI:** 10.1038/s41598-018-28579-4

**Published:** 2018-07-17

**Authors:** Judith J. M. Ceelen, Annemie M. W. J. Schols, Anita E. M. Kneppers, Roger P. H. A. Rosenbrand, Magda M. Drożdż, Stefan J. van Hoof, Chiel C. de Theije, Marco C. J. M. Kelders, Frank Verhaegen, Ramon C. J. Langen

**Affiliations:** 1 Department of Respiratory Medicine, Maastricht University Medical Center, Maastricht, The Netherlands; 2 Department of Radiation Oncology (MaastRO), Maastricht University Medical Center, Maastricht, The Netherlands

## Abstract

Exacerbations in Chronic obstructive pulmonary disease (COPD) are often accompanied by pulmonary and systemic inflammation, and are associated with an increased susceptibility to weight loss and muscle wasting. As the emphysematous phenotype in COPD appears prone to skeletal muscle wasting, the aims of this study were to evaluate in emphysematous compared to control mice following repetitive exacerbations (1) changes in muscle mass and strength and, (2) whether muscle mass recovery and its underlying processes are impaired. Emphysema was induced by intra-tracheal (IT) elastase instillations, followed by three weekly IT-LPS instillations to mimic repetitive exacerbations. Loss of muscle mass and strength were measured, and related to analyses of muscle protein turnover and myogenesis signaling in tissue collected during and following recovery. Emphysematous mice showed impaired muscle mass recovery in response to pulmonary inflammation-induced muscle atrophy. Proteolysis and protein synthesis signaling remained significantly higher in emphysematous mice during recovery from LPS. Myogenic signaling in skeletal muscle was altered, and fusion capacity of cultured muscle cells treated with plasma derived from LPS-treated emphysematous mice was significantly decreased. **In conclusion**, repetitive cycles of pulmonary inflammation elicit sustained muscle wasting in emphysematous mice due to impaired muscle mass recovery, which is accompanied by aberrant myogenesis.

## Introduction

Chronic obstructive pulmonary disease (COPD) is characterized by persistent airflow obstruction, inflammation and remodeling of the airways, which may include development of emphysema^1^. A major extra-pulmonary comorbidity of COPD is muscle wasting, which has been shown to be an independent predictor of exercise performance^2^ and of mortality^3^. COPD patients with an emphysematous phenotype appear to be more prone to skeletal muscle wasting^4^.

Disease severity of COPD is not only characterized by the degree of airflow obstruction or emphysema but also by acute flare-ups of disease symptoms, referred to as exacerbations. Frequent exacerbations accelerate disease progression and decrease survival^5^. Furthermore, Alahmari *et al*. showed that frequent exacerbations are associated with increased muscle weakness and a decline in exercise capacity^6^. Importantly, COPD patients suffering from frequent exacerbations are more prone to weight loss and muscle wasting^7,8^. However, whether emphysematous and frequent exacerbation phenotypes synergistically predispose to -or elicit- muscle wasting is currently unknown.

Exacerbations can be triggered by bacterial respiratory infections^9^ and are often accompanied by pulmonary and systemic inflammation^10,11^. Previous studies have shown that pulmonary inflammation evoked by the bacterial component LPS, is sufficient to induce an acute^12,13^, but reversible^14^ loss of skeletal muscle mass, which is the result of increased muscle proteolysis and decreased protein synthesis. In contrast, continuous pulmonary inflammation in presence of emphysema as observed in SP-C/TNF transgenic mice is accompanied by a sustained reduction in body and muscle weight. In addition, impaired muscle regeneration has been documented in these mice, suggesting that emphysema or chronic inflammation may impair the recovery of atrophied muscle^15^. This suggests that muscle atrophy in COPD may follow a stepwise pattern in which each exacerbation causes an acute decrease in muscle mass, followed by impaired muscle regeneration in the stable clinical condition due to interference with protein synthesis and myogenesis due to the underlying disease.

However, the effect of transient pulmonary inflammation in presence of emphysema (i.e. exacerbation) on skeletal muscle mass recovery has not been addressed. In addition, the cumulative effects of repetitive exacerbations on muscle mass maintenance and muscle protein turnover regulation have not been studied. As we postulate that skeletal muscle atrophy in COPD is the consequence of active loss of muscle mass during disease exacerbations and impaired muscle re-growth during stable disease, we tested the hypothesis that repetitive pulmonary inflammation in emphysematous mice evokes sustained muscle atrophy resulting from impaired muscle mass recovery. Thus, a model in which intratracheal instillation of LPS evokes exacerbation-like pulmonary inflammation in mice with elastase-induced emphysema^16^, was adapted to longitudinally monitor muscle mass and strength recovery in response to repetitive pulmonary inflammation. Mice received intratracheal (IT) instillations with elastase in order to induce emphysema, followed by three consecutive weekly IT instillations with LPS administration to mimic exacerbations. Using this model, our first objective was to evaluate the degree of muscle wasting after repetitive exacerbations in emphysematous mice. Changes in muscle mass and strength were evaluated non-invasively using grip strength measurements and CT-scan analysis on multiple time points throughout the experiment. To evaluate the potential contribution of altered protein turnover regulation and myogenesis in emphysematous mice, markers of these processes were measured during the muscle mass recovery phase following pulmonary inflammation as the second objective of this study.

## Results

### Development of emphysema, pulmonary and systemic inflammation

**Figure 1:**
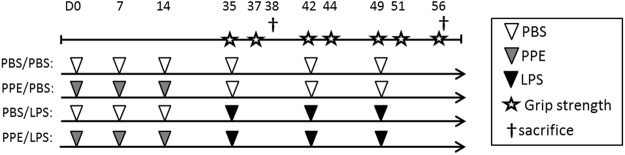
Experimental set-up. Mice were intratracheally instilled (IT) with elastase to induce emphysema or PBS (vehicle control, vc), followed by three weekly instillations with LPS or vc. 3 days after the 1^st^ IT-LPS (D38) or 7 days after the 3^rd^ LPS (D56) gastrocnemius muscle was collected for evaluation of protein turnover signaling. D = day of experiment.

**Figure 2:**
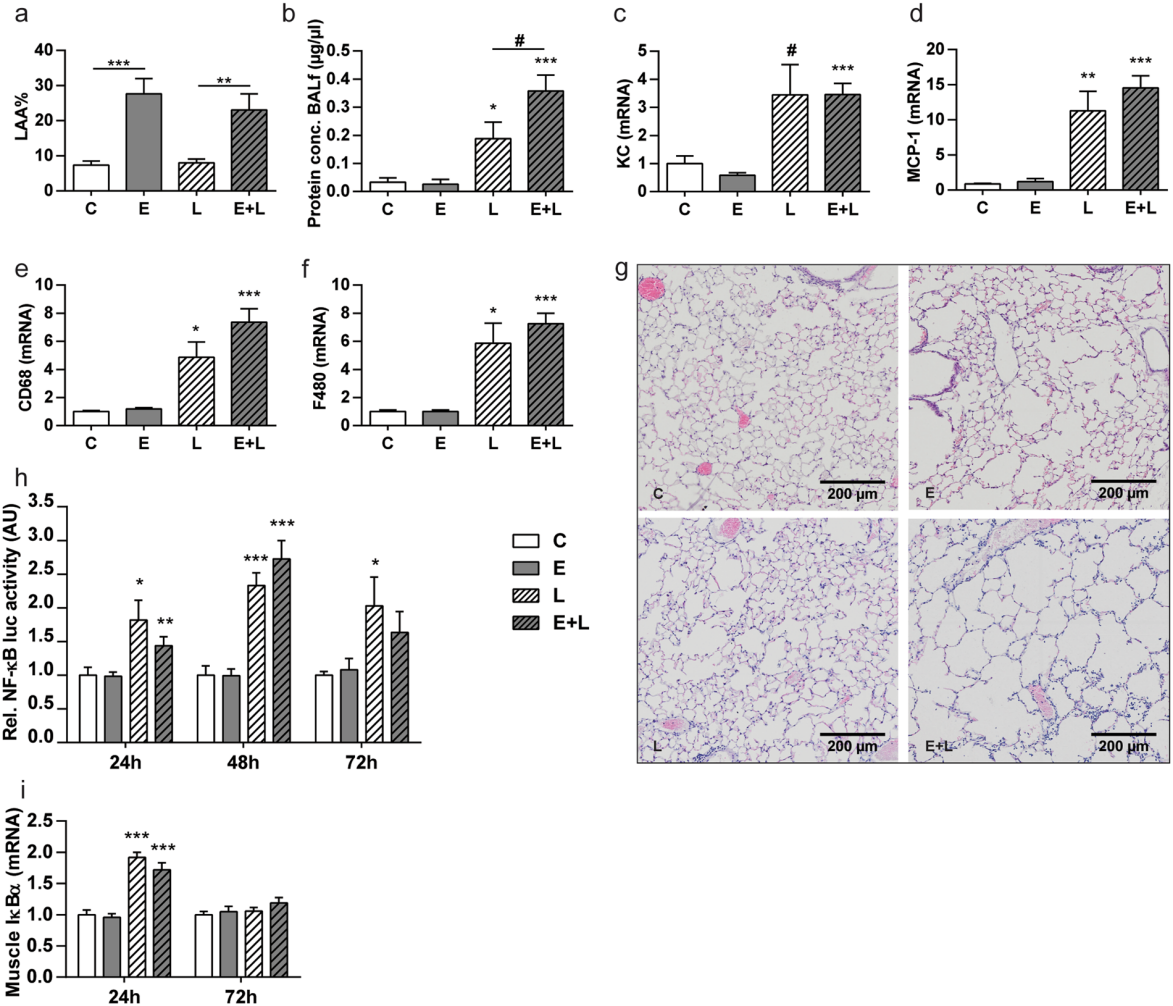
Development of emphysema and pulmonary and systemic inflammation after intra-tracheal instillation with elastase and LPS, respectively. Mice were intratracheally instilled with elastase to induce emphysema or vc. (**a**) Changes in LAA% 21 days after the 3^rd^ IT-elastase, as determined by µCT scan analysis. n = 16 per group. Then mice received a single bolus of LPS or vc. 72 h after LPS or vc instillation (**b**) BALf was collected and total protein concentration was determined. Lungs were collected for (**g**) histological evaluation or assessment of mRNA abundance of (**c**) KC, (**d**) MCP-1, (**e**) CD68 and (**f**) F480, normalized to GeNorm and expressed as fold change compared to control. n = 4–8 per group. (**h**) BALf was collected from mice 24 h, 48 h and 72 h after LPS or vc instillation. Cells isolated from BALf were used to produce conditioned medium. NF-κB luciferase activity was measured in lysates prepared from C2C12 myotubes after stimulation with conditioned medium. n = 5–12 per group. i) 24 h and 72 h after LPS gastrocnemius muscle was collected for evaluation of mRNA abundance of IκBα, normalized to GeNorm and expressed as fold change compared to control. n = 7–8 per group. Significant differences were determined using an independent-samples T-test. Symbols above a bar refer to a comparison with the respective control (L vs. C and E + L vs. E). A symbol above a line refers to a comparison between the bars indicated by the line. *p < 0.05, **p < 0.01, ***p < 0.001. ^#^represents a trend.

Mice were subjected to the experimental protocol as depicted in Fig. 1. To non-invasively determine the development of emphysema in response to intratracheal elastase, µCT scans were made and analyzed, and the percentage of low attenuation area (LAA%) was calculated for each lung. Mice that received elastase had a significantly higher LAA% than the control mice (Fig. 2a), confirming the presence of emphysema prior to LPS or vehicle instillations. Histological analyses of lung sections post-LPS or –vc administration confirmed airspace enlargement in elastase-treated mice (Fig. 2g) as shown previously in this model^14,16^, which based on the measured LAA% is expected to correspond with a mean linear intercept of 45–50^14^, and is similar to the extent of emphysema observed following chronic smoke exposure in mice^17^. Broncho-alveolar lavage (BAL)fluid protein content levels were increased following LPS, indicative of lung injury in control and emphysematous mice (Fig. 2b).

To verify whether pulmonary inflammation was induced in response to IT-LPS, expression levels of several chemokines and macrophage markers were determined in lung tissue. Control and emphysematous mice both showed increased levels of KC, MCP-1, CD68, and F480 expression post IT-LPS, indicating a similar extent and composition of the lung inflammatory response (Fig. 2c–f).

To assess the potential systemic ramifications of the acute lung inflammatory response, *in vitro* cultured skeletal muscle cells were incubated in presence of media conditioned by cells isolated from BALf (Fig. 2h). NF-κB activity was induced in presence of CM derived from IT-LPS treated (24 h, 48 h and 72 h) control and emphysematous mice. In line, a transient increase of IκBα mRNA indicative of NF-κB activation was observed in skeletal muscle of LPS-treated control and emphysematous mice (Fig. 2i).

### Decreased muscle strength and mass following repetitive pulmonary inflammation in emphysematous mice

**Figure 3:**
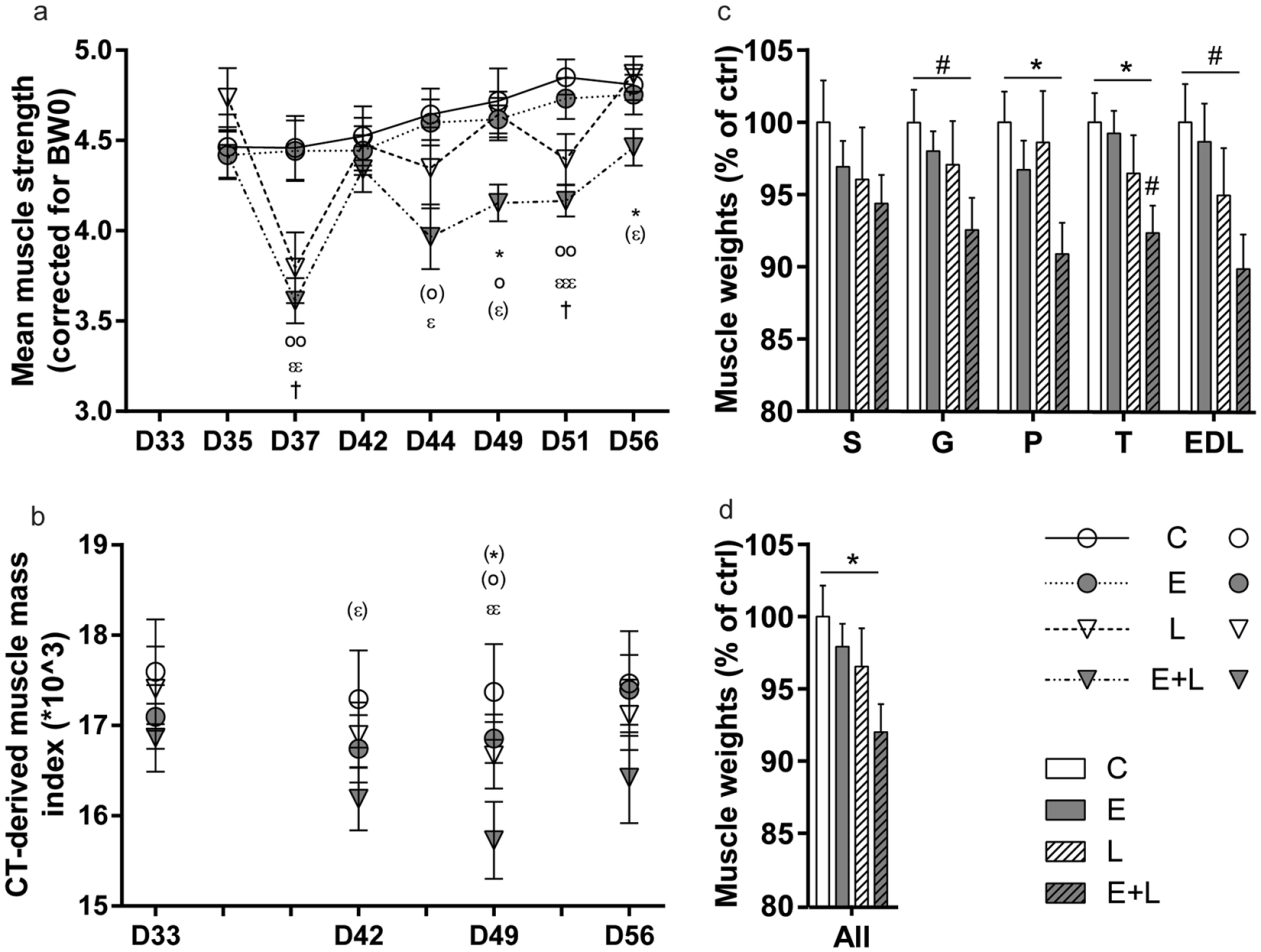
Decreased muscle strength and mass following repetitive pulmonary inflammation in emphysematous mice. Mice were intratracheally instilled with elastase to induce emphysema or vc, followed by three weekly instillations with LPS or vc. (**a**) Mean grip strength, corrected for starting body weight, was determined right before each IT-LPS (D35, D42, D49), 2 days after each IT-LPS (D37, D44, D51) and 7 days after the third IT-LPS (D56). *p*-values were calculated using a repeated measures two-way ANOVA. (**b**) CT-derived muscle mass corrected for bodyweight D0. (**c**) 7 days (D56) after the third IT-LPS, mice were sacrificed and skeletal muscle wet weights of the soleus (S), gastrocnemius (G), plantaris (P), tibialis (T) and EDL were measured and corrected for tibia length. (**d**) All skeletal muscle wet weights combined. n = 6–13. Significant differences were determined using a one-way ANOVA. For a and b: ‘†’ indicates C vs. L, ‘o’ indicates E vs. E + L, ‘ε’ represents C vs. E + L and *represents L vs. E + L. For c and d: Symbols above a bar refer to a comparison with the respective control (L vs. C and E + L vs. E). A symbol above a line refers to a comparison between the bars indicated by the line. *p < 0.05, **p < 0.01, ***p < 0.001. ^#^or symbols between brackets represent a trend.

Bodyweights were indiscernible between control and emphysematous mice prior to induction of pulmonary inflammation, and IT-LPS administration resulted in a similar, transient reduction of bodyweight in both groups (Supplementary Fig. S1). 48 h (D37) following induction of pulmonary inflammation, a rapid decrease in skeletal muscle strength (Fig. 3a) occurred, which was similar in healthy and emphysematous mice. Although recovery of muscle strength 1 week (D42) following the first LPS instillation was not different compared to vc (Fig. 3a), muscle mass determined by micro-CT was not returned to control in emphysematous mice 1 week (D42) following pulmonary inflammation (Fig. 3b). Repetitive exacerbations, simulated by multiple rounds of LPS administration, revealed complete recovery of muscle force (Fig. 3a) and mass determined by micro-CT (Fig. 3b) or muscle wet weight (Fig. 3c) of control mice. In contrast, longitudinal determination of muscle strength (Fig. 3a) or mass (Fig. 3b) of emphysematous mice consistently revealed significant decreases or trends 1 week following the second and third LPS instillation (D49 and D56 respectively). This impaired muscle mass recovery was confirmed by the sustained reduction in skeletal muscle wet weight, only observed in emphysematous mice subjected to repetitive pulmonary inflammation (Fig. 3c,d).

### No sustained alterations in regulation of muscle protein turnover signaling in emphysematous mice after recovery from repetitive pulmonary inflammation

Next, we determined whether the lack of recovery in muscle mass and strength in E + L mice may be the consequence of changes in protein turnover signaling which were sustained 7 days following the third IT-LPS administration. Expression levels of several effectors of ubiquitin 26S-proteasome (UPS)-mediated and autophagy lysosomal pathway (ALP)-mediated proteolysis (Supplementary Fig. S2), protein synthesis (Supplementary Fig. S3), as well as regulatory kinases and transcriptional factors of these processes (Supplementary Fig. S4) were measured in gastrocnemius muscle. Combined, these analyses showed that the impaired recovery of muscle mass and strength of emphysematous mice after repetitive pulmonary inflammation cannot be attributed to sustained differences in regulation of proteolysis or protein synthesis signaling.

### Subtle increases in muscle protein turnover signaling in emphysematous mice during recovery from pulmonary inflammation

**Figure 4:**
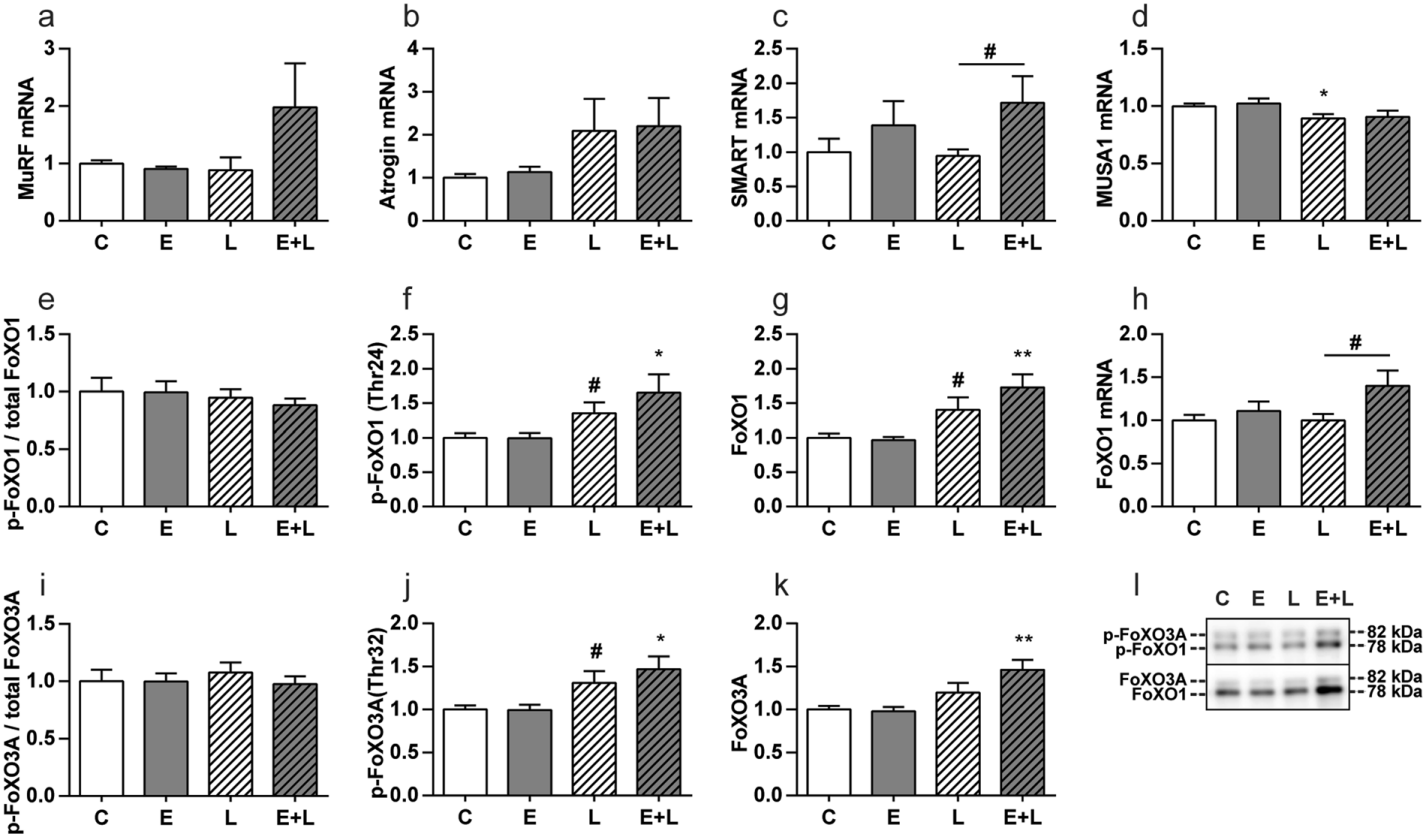
UPS signaling in skeletal muscle is only slightly increased in emphysematous mice during recovery following pulmonary inflammation. Mice were intratracheally instilled with elastase to induce emphysema or vc, followed by a single bolus of LPS or vc. 72 hours after LPS gastrocnemius muscle was collected for evaluation of mRNA abundance of (**a**) MuRF1, (**b**) Atrogin-1, (**c**) SMART, (**d**) MUSA1 and (**h**) FoXO1, normalized to GeNorm and expressed as fold change compared to control. Alternatively, protein levels of (**f**) phosphorylated FoXO1 (Thr24), (**g**) total FoXO1, (**j**) phosphorylated FoXO3A (Thr32) and (**k**) total FoXO3A were assessed. (**l**) Representative western blots of indicated proteins. Uncropped western blots are shown in Supplementary Fig. S4. Ratio of (**e**) phosphorylated FoXO1 over total FoXO1 and (**i**) phosphorylated FoXO3A over total FoXO3A. n = 7–8 per group. Significant differences were determined using an independent-samples T-test. Symbols above a bar refer to a comparison with the respective control (L vs. C and E + L vs. E). A symbol above a line refers to a comparison between the bars indicated by the line. *p < 0.05, **p < 0.01, ***p < 0.001. ^#^represents a trend.

**Figure 5:**
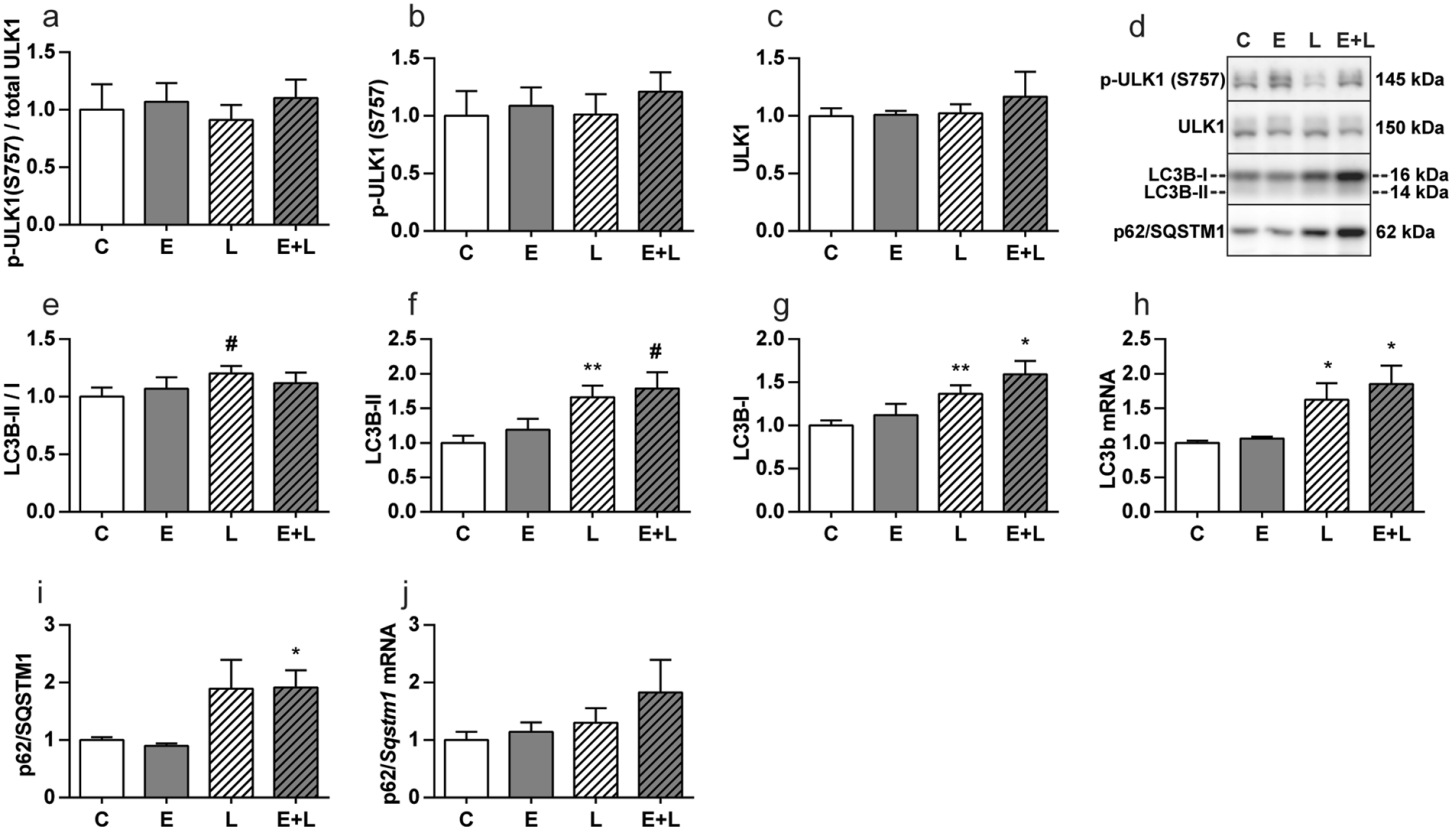
Markers of downstream ALP signaling in skeletal muscle are slightly increased in control and emphysematous mice during muscle recovery from pulmonary inflammation. Mice were intratracheally instilled with elastase to induce emphysema or vc, followed by a single bolus of LPS or vc. 72 hours after LPS gastrocnemius muscle was collected. Protein levels of (**b**) phosphorylated ULK1 (S757), (**c**) total ULK1, (**f**) LC3B-II, (**g**) LC3B-I and (**i**) p62 were assessed in lysates of gastrocnemius muscle tissue with western blot analysis. (**d**) Representative western blots of indicated proteins. Uncropped western blots are shown in Supplementary Fig. S5. Ratio of (**a**) phosphorylated ULK1 over total ULK1 and (**e**) LC3B-II over LC3B-I. mRNA transcript levels of (**h**) LC3B and (**j**) p62 were determined, normalized to geNorm, and expressed as fold change compared to control. n = 7–8 per group. Significant differences were determined using an independent-samples T-test. Symbols above a bar refer to a comparison with the respective control (L vs. C and E + L vs. E). A symbol above a line refers to a comparison between the bars indicated by the line. *p < 0.05, **p < 0.01, ***p < 0.001. ^#^represents a trend.

To determine whether protein turnover signaling was impaired in emphysematous mice during the early phase of the muscle mass recovery period, effectors and regulators of proteolysis and synthesis signaling were determined 72 hours after IT-LPS instillation. Activation and involvement of effectors of UPS-mediated proteolysis during muscle atrophy acutely after pulmonary inflammation, has been demonstrated previously^12,13^, and is similar in emphysematous and control mice 48 h post IT-LPS^14^. Expression of the E3 Ub-ligases (Fig. 4a–d) was no longer significantly elevated. Correspondingly, inhibitory phosphorylation (Fig. 4f,j) levels of FoXO1 and FoXO3A were increased in emphysematous mice after IT-LPS, in presence of increased FoXO1 mRNA and FoXO1 and FoXO3A total protein abundance (Fig. 4g,h,k). Evidence suggestive of ALP-mediated proteolysis during muscle atrophy in response to acute pulmonary inflammation, has been described previously, and is similar in emphysematous and control mice 48 h post IT-LPS^14^. To study ALP regulation during recovery, several markers of the ALP were measured. Protein levels of total and phosphorylated ULK1 were not changed between any of the conditions (Fig. 5a–c), indicating the absence of upstream ALP-activating stimuli in this phase of muscle mass recovery. To facilitate autophagy the cytosolic form of LC3 (LC3B-I) is conjugated to the lipidated form (LC3B-II), resulting in recruitment to the autophagosomal membrane^18^. Although the LC3B-II/LC3B-I ratio was not affected (Fig. 5e), LC3B-I and –II protein, and LC3 transcript levels were still increased in control and emphysematous mice after LPS (Fig. 5f–h). Increases in p62 following LPS were only significant for p62 protein in emphysematous mice (Fig. 5i,j).

**Figure 6:**
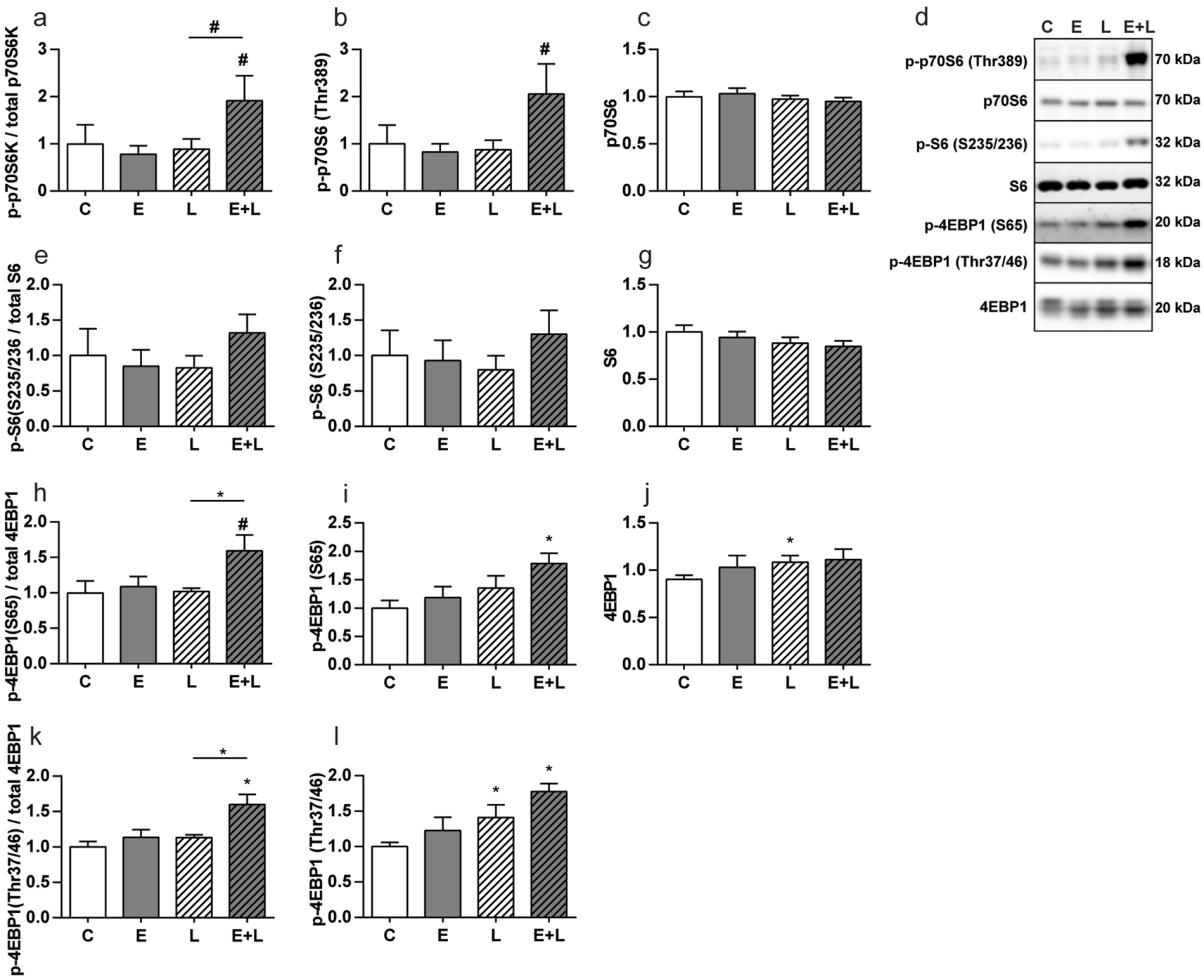
Increased protein synthesis signaling in skeletal muscle of emphysematous but not control mice during recovery from pulmonary inflammation. Mice were intratracheally instilled with elastase to induce emphysema or vc, followed by a single bolus of LPS or vc. 72 hours after LPS gastrocnemius muscle was collected. Protein levels of (**b**) phosphorylated p70S6 (Thr389), **(c**) total p70S6, (**f**) phosphorylated S6 (ser235/236), (**g**) total S6, (**i**) phosphorylated 4EBP1(ser65) and (**l**) 4EBP1(Thr37/46) and (**j**) total 4EBP1 were assessed in lysates of gastrocnemius muscle tissue with western blot analysis. (**d**) Representative western blots of indicated proteins. Uncropped western blots are shown in Supplementary Fig. S6. Ratio of (**a**) phosphorylated p70S6 over total p70S6, (**e**) phosphorylated S6 over total S6 and (**h**) phosphorylated 4EBP1 (ser65) and (**k**) phosphorylated 4EBP1 (Thr37/46) over total 4EBP1. n = 7–8 per group. Significant differences were determined using an independent-samples T-test. Symbols above a bar refer to a comparison with the respective control (L vs. C and E + L vs. E). A symbol above a line refers to a comparison between the bars indicated by the line. *p < 0.05, **p < 0.01, ***p < 0.001, ^#^represents a trend.

Previous work demonstrates a strong inhibition of protein synthesis signaling in skeletal muscle 48 h following induction of pulmonary inflammation, which is similar in emphysematous and control mice^14^. To investigate whether protein synthesis was altered during recovery of inflammation-induced atrophy, protein abundance and activating and inhibitory phosphorylation levels of p70S6K, S6 and 4EBP1 were determined (Fig. 6). Phosphorylation levels of p70S6K and 4EBP1 (both Ser65 and Thr37/46) were significantly increased in emphysematous mice only after LPS. Combined, these data revealed small but consistent differences in protein turnover signaling in emphysematous mice 72 hours following induction of pulmonary inflammation, which may reflect altered kinetics of processes involved in muscle mass recovery.

### Myogenic signaling and fusion is affected in emphysematous mice during recovery from pulmonary inflammation

**Figure 7:**
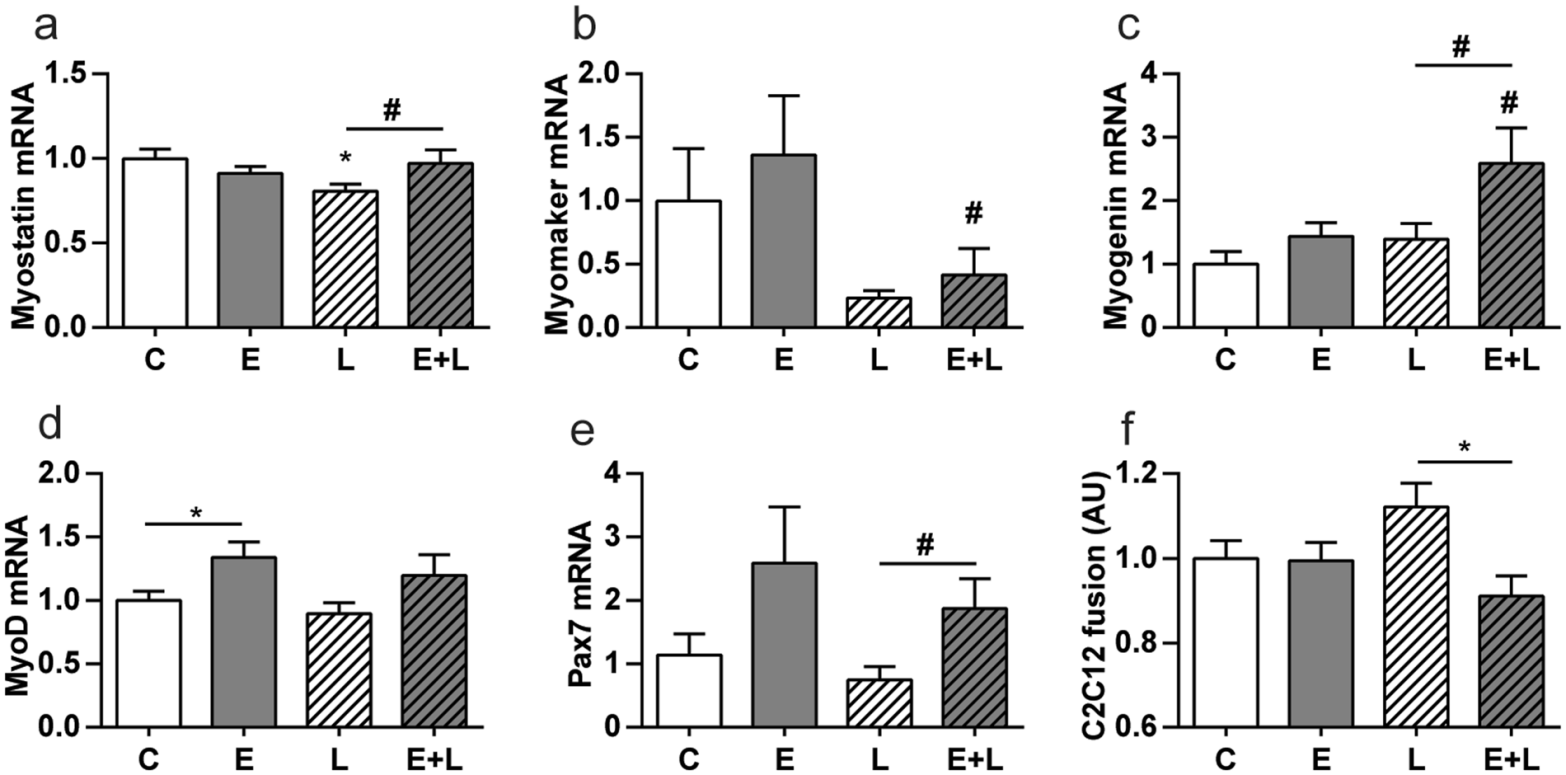
Altered myogenic signaling in skeletal muscle and reduced myogenic fusion in the presence of plasma from LPS-treated emphysematous mice. Mice were intratracheally instilled with elastase to induce emphysema or vc, followed by a single bolus of LPS or vc. 72 hours after LPS gastrocnemius muscle was collected for evaluation of mRNA abundance of (**a**) Myostatin, (**b**) Myomaker, (**c**) Myogenin, (**d**) MyoD and (**e**) Pax7 normalized to GeNorm and expressed as fold change compared to control. n = 7–8 per group. *p < 0.05, **p < 0.01, ***p < 0.001. ^#^Represents a trend. Alternatively, **(e)** plasma was collected for evaluation of properties affecting myogenic fusion. To this end, LV-Floxed luc myoblasts were added to differentiated CRE-C2C12 myotubes as described in detail elsewhere^49^ and incubated with 5% plasma for 3 days. Cells were harvested and cell fusion-dependent luciferase was measured. n = 10 per group. Significant differences were determined using an independent-samples T-test. Symbols above a bar refer to a comparison with the respective control (L vs. C and E + L vs. E). A symbol above a line refers to a comparison between the bars indicated by the line. *p < 0.05, ^#^represents a trend.

There is ample evidence implying myogenesis during muscle mass recovery following atrophy^15,19,20^. To determine whether myogenesis signaling is affected in emphysematous mice after pulmonary inflammation-induced atrophy, several markers of myogenesis were measured in gastrocnemius muscle. Although assessment of protein abundance would be preferential, limitations in the specificity and sensitivity of commercially available antibodies in our hands prompted us to evaluate mRNA transcript levels instead for a comprehensive analysis of the expression of myogenic regulatory factors. mRNA levels of Myostatin, an inhibitor of myogenesis, were slightly increased in emphysematous compared to control mice following LPS (Fig. 7a). Similarly, mRNA transcript levels of Myomaker, Myogenin, and Pax7, which represent markers of myogenic fusion, late differentiation and satellite cell number^21–25^, were also slightly increased in emphysematous compared to normal mice following LPS-treatment (Fig. 7b,c,e), while MyoD expression was already altered in emphysematous mice prior to pulmonary inflammation (Fig. 7d).

Myogenesis is subject to paracrine and endocrine regulation by hormones, myokines and cytokines^26–28^. We next determined whether the remarkable differences in expression of myogenesis markers may reflect a response to an altered endocrine milieu, and coincide with altered myonuclear accretion as the final step of myogenesis involvement in muscle regeneration^29^. Using a model of postnatal myonuclear accretion, the capacity of myoblasts to fuse with differentiated C2C12 myotubes was assessed in presence of diluted plasma obtained from control or emphysematous mice, following LPS or vc. No difference in myonuclear accretion was observed in presence of plasma of control or emphysematous mice that were not subjected to LPS. In contrast, cell cultures incubated with plasma from emphysematous mice after pulmonary inflammation showed a significant reduction in fusion capacity compared to myoblast-myotube co-cultures treated with plasma from LPS-treated control mice (Fig. 7f).

## Discussion

The effects of frequent exacerbations on skeletal muscle in COPD are not known. Specifically, the cumulative effects of multiple exacerbations on muscle mass, and consequences of exacerbations on muscle recovery from atrophy including the myocellular processes in control of muscle mass are not thoroughly understood. In this study, an experimental mouse model was implemented in which control or emphysematous mice were administered multiple intratracheal LPS (IT-LPS) instillations to simulate repetitive exacerbations. Three consecutive cycles of pulmonary inflammation resulted in sustained muscle atrophy in emphysematous but not control mice. Repetitive exacerbations were not accompanied by sustained differences in protein synthesis or proteolysis signaling between control and emphysematous mice. In contrast, protein turnover signaling during muscle mass recovery was increased, which was associated with altered myogenesis and a reduced myogenic fusion-supportive endocrine milieu.

Emphysematous mice did not show reduced muscle mass or strength which is line with previous reports in similar models^30^. Conversely, Fermoselle *et al*. showed that body weight and gastrocnemius mass was significantly less in emphysematous mice compared to controls^31^. However, those mice were still growing during the experiment, suggesting that emphysema may have had an attenuating effect on muscle growth as part of post-natal development. In smoke-induced emphysema body weight, and to a lesser extent muscle weight, were reduced^32^, which may be a consequence of a more pronounced chronic pulmonary inflammation in that model.

Our data reveal that following repetitive pulmonary inflammation, there is sustained muscle wasting in emphysematous mice. This is not the result of an increased acute effect of LPS, since the LPS-induced, acute decreases in grip strength are not different between emphysematous and control mice. In line with this, it has been shown previously that acute inflammation-induced muscle atrophy in emphysematous mice is not more extensive than in healthy mice^14^. Consequently, the sustained muscle wasting in emphysematous mice following repetitive cycles of LPS is not the cumulative effect of more extensive acute losses of muscle mass. Instead, the delayed restoration of muscle mass and strength in E + L mice, suggests an impaired ability to recover from muscle atrophy, resulting in a stepwise decrease of muscle mass in emphysematous mice.

Previously it has been shown that changes in muscle mass are accompanied by alterations in protein turnover signaling in response to pulmonary^13,14^ or systemic inflammation in mice^33,34^. Increased proteolysis signaling, including markers suggestive of UPS and ALP activation during acute muscle atrophy following pulmonary inflammation has been well documented in control^12,13^ and emphysematous mice^14^, but appears to be of transient nature as UPS and ALP activity have returned to or were below baseline level 7 days following the last IT-LPS instillation. Of note, 26S-proteasomal degradation rates and autophagy flux measurements should complement the UPS and ALP marker analyses to confirm normalized activity of muscle proteolysis. Similarly, protein synthesis signaling has returned to baseline levels in both control and emphysematous one week after repetitive pulmonary inflammation, including the reduction in S6 phosphorylation levels in emphysematous mice described previously^14^. Combined, these data indicate that a persistent imbalance in protein turnover signaling is not the basis of sustained muscle atrophy following repetitive pulmonary inflammation in emphysematous mice.

Skeletal muscle mass recovery upon the termination of an atrophying stimulus is accompanied by temporal reductions in proteolytic activity, activation of protein synthesis and myogenic signaling^20^. In a previous study, we monitored changes in protein synthesis signaling and proteolysis regulation throughout the complete timeframe of pulmonary inflammation-induced muscle mass loss and recovery^35^. Based on that work, 3 days after induction of pulmonary inflammation was identified as a turning point at which proteolysis signaling was decreased to baseline, and markers for protein synthesis started to increase. In line with this, proteolysis signaling, i.e. markers of UPS and ALP, have returned to baseline at this time point in healthy LPS-treated mice. In contrast, these markers remained significantly higher in E + L mice, suggesting extended proteolysis signaling in skeletal muscle of emphysematous mice following pulmonary inflammation.

Multiple studies have shown that muscle regrowth following atrophy is accompanied by a reactivation of suppressed protein synthesis signaling^20,36,37^. As expected, markers of protein synthesis returned to baseline in LPS-treated mice upon initiation of muscle mass recovery. However, in emphysematous mice synthesis signaling was upregulated at 72 hours post LPS. This may reflect a futile compensatory response to counteract extended proteolysis signaling, which accompanies delayed muscle mass recovery. Interestingly, increased protein synthesis signaling in atrophied skeletal muscle has been described in patients with COPD^38^.

Since myogenesis is activated during muscle growth and regrowth following atrophy^20^, impaired signaling in this process might explain differences that were seen in recovery of muscle mass and strength after LPS between emphysematous and healthy mice. Myogenic signaling has not previously been documented upon recovery from pulmonary inflammation induced atrophy, but muscle reloading following disuse-atrophy is accompanied by satellite cell activation and proliferation, and increased markers of myogenic differentiation and fusion^20^. Although the differences in markers for myogenesis signaling during recovery from pulmonary inflammation between control and emphysematous mice are subtle, sustained impairment of this regenerative response may adversely affect muscle mass, in particular after repetitive cycles of atrophy and muscle mass recovery. Similarly, in response to chronic pulmonary inflammation, impaired muscle mass recovery is accompanied by systemic inflammation and aberrant myogenic signaling^15^.

Myogenesis is dictated by local and endocrine mediators^26–28^. The reduced myonuclear accretion of myotube-myoblast co-cultures in presence of plasma of E + L mice, suggests impairments in the endocrine milieu of emphysematous mice following pulmonary inflammation to support this final step of myogenesis during muscle mass recovery. Although this reduced myonuclear accretion will have to be confirmed *in vivo* in future work by assessing incorporation of labeled satellite cells into myofibers^39^, our findings are in line with Corrick *et al*., showing that myogenesis can be influenced by circulating factors. They found that when differentiating cells were incubated with plasma from burn victims, myogenic fusion signaling was reduced and myogenesis impaired^40^. Interestingly, a robust increase in circulating inflammatory factors was present in these sera, and previously it has been shown that pro-inflammatory cytokines can inhibit myogenic differentiation. TNFα or IL-1 when administered to cultured differentiating myoblasts, inhibit myogenic differentiation^41,42^. Changes in levels of circulating cytokines due to pulmonary inflammation over time have not been investigated in this study. However, as previously reported, circulating levels of pro-inflammatory cytokines are increased up to 2 days following induction of pulmonary inflammation in this model^13^. Possibly, impaired aberrant myogenic signaling in muscle of emphysematous mice might be due to sustained high levels of pro-inflammatory cytokines.

Alternatively, the expression pattern of anti-inflammatory cytokines remains to be determined in this model, but IL-15 has been implicated in the resolution of (pulmonary) inflammation. Interestingly, these beneficial effects of anti-inflammatory cytokines may extend beyond the lung, as IL-15 has been shown to stimulate myogenic differentiation^43^ and increase accretion of myosin heavy chains in primary human skeletal muscle cells^44^. Accordingly, a rise in systemic levels of anti-inflammatory cytokines may stimulate muscle myogenic signaling, while a delay may culminate in impaired muscle recovery. However, whether these cytokine levels are temporarily affected in the circulation of emphysematous mice following pulmonary inflammation, and are responsible for the altered response in turnover and myogenic signaling remains to be resolved.

In conclusion, repetitive cycles of pulmonary inflammation elicit sustained muscle wasting in emphysematous mice compared to LPS-treated healthy mice. This is due to impaired recovery rather than more extensive acute pulmonary inflammation-induced atrophy. The impaired restoration of muscle mass and strength could not be attributed to persistent changes in protein turnover regulation. Instead, the observation that factors present in the circulation interfered with fusion of cultured muscle cells combined with altered expression of mediators of myogenesis in muscle tissue, suggests that impaired recovery of muscle mass in emphysematous mice following repetitive pulmonary inflammation is due to impaired muscle regenerative capacity.

## Methods

### Animals and experimental protocol

This study was approved by the Animal Experimental Committee of Maastricht University and carried out in accordance with the guidelines and regulations. Experimental emphysema can be evoked in mice by chronic cigarette smoke exposure or elastase instillation^45^. The development of smoke-induced emphysema requires long-term exposure and is a laborious procedure. In addition, smoke exposure also induces chronic pulmonary inflammation^45^. Since our goal was to model repetitive pulmonary inflammation-induced exacerbations in an emphysematous context, we chose elastase-induced emphysema as a model as the emphysema is accompanied by less pronounced pulmonary inflammation compared to the smoke model^16^. This allowed studying the systemic consequences of exacerbations evoked by transient pulmonary inflammation in the context of emphysema. Twelve-week-old C57BL/6 J male mice (Charles River Laboratories International, Wilmington, MA) (n = 92) were randomly divided into four groups: control (C), elastase-treated (E), LPS-treated (L) and both elastase- and LPS-treated (E + L). Animals received 3 weekly intratracheal instillations (D0, D7, D14) with 4 U of porcine pancreatic elastase (PPE) (Wako Instruchemie BV, Delfzijl, Netherlands) dissolved in 50 µl sterile PBS or 50 µl of PBS alone (vehicle control, vc). 21 days after the last elastase instillation, degree of emphysema was verified by µCT-scan analysis as was described previously^14^. Then mice received 3 weekly intratracheal instillations (D35, D42, D49) with 2.5 µg LPS per gram mouse (*Escherichia coli*, serotype O55:B5; Sigma, St. Louis, MO) dissolved in 50 µl sterile 0.9% NaCl or 50 µl sterile 0.9% NaCl alone (vc). Previous research by our group determined 72 hours following pulmonary inflammation as a turning point at which proteolysis signaling becomes downregulated and protein synthesis is activated to support recovery of atrophied muscle in healthy mice. Therefore, mice were sacrificed 3 days after the first IT-LPS to assess lung inflammation (D38) and 7 days after the third IT-LPS (D56) to assess muscle atrophy. At both time points markers for protein turnover signaling and myogenesis were assessed in skeletal muscle. The lungs were collected and stored at −80 °C for RNA extraction. Blood was collected from the vena cava to obtain plasma. The soleus (S), gastrocnemius (G), tibialis anterior (T), plantaris (P), and EDL (E) muscles were collected from both hind limbs, using standardized dissection methods and stored at −80 °C for RNA and protein extraction. For a subset of analyses concerning the kinetics of the inflammatory response, tissue and material obtained from a previous study^14^ was used, which was collected 24 h(D36) or 48 h(D37) after the first IT-LPS or vc instillation.

### Assessment of lung inflammation

BALfluid which was collected 72 h post LPS was centrifugated (5 min at 1500 rpm, 4 °C), pelleted cells were resuspended in differentiation medium (i.e., Dulbecco’s Modified Eagle’s Medium containing 0.5% FBS, 50 U/ml penicillin, and 50 mg/ml streptomycin) and incubated overnight at 37 °C to obtain conditioned medium (CM). CM was centrifugated and the supernatant was stored at −80 °C until further use. NF-κB luc-reporter cells^42^ were grown on Matrigel (Becton Dickinson Labware, Bedford, MA) and differentiated into myotubes (as described previously^46^. Myotubes were incubated with CM for 8 hours at 37 °C, whereafter they were harvested in Reporter lysis buffer (Promega, Madison, USA). Luminescence was determined using a luminometer (Berthold Lumat LB 9507, Belgium).

### µCT imaging and analysis

Mice were anesthetized with a mixture of air and 4% isoflurane and scanned using a cone beam, µCT scanner (XRAD-225Cx, Precision X-Ray, North Branford, USA) at a dose of 0.28 Gy. µCT images were acquired at 80 kVp and reconstructed to a 3D image volume with an isotropic voxel spacing of 0.2 mm. Resulting image data were analyzed using SmART-Plan (version 1.3.6)^47^. A selection of the lungs was made and the density of each voxel was plotted in a density histogram. The LAA threshold was set at −426 Hounsfield units (HU). The LAA% was calculated as the ratio of LAA to the total lung area. The muscle of the hindlimbs was delineated using SmART-Plan. Muscle volume was determined as the total selected volume minus air and bone (thresholds −460 and 520 HU respectively). Muscle volumes were then converted by using CT to mass density calibration function, established using the same imaging protocol. The HU to density calibration was described previously^14^.

### *In vivo* muscle strength

Forelimb grip strength was measured with a calibrated grip strength tester (Panlab, Cornella, Spain) by allowing the mouse to grasp and pull the trapeze bar with both forelimbs while holding the mouse by the tail, parallel to orientation of the meter. For each measurement, a set of 5 repetitions was performed. For mean grip strength these 5 repetitions were averaged, and for maximal grip strength the maximal tension recorded was used.

### RNA Isolation

RNA was isolated from homogenized gastrocnemius muscle from mice sacrificed 7 days after the third IT-LPS using the RNeasy plus mini kit (Qiagen). cDNA was made with the Tetro cDNA Synthesis kit (GC biotech). Primer sequences of transcripts of interest are provided in Supplementary Table S1. The relative DNA starting quantities of the samples were derived using LinRegPCR software (Version 2014.0, Ruijter). The expression of genes of interest was normalized to the geometric average of three or four reference genes (cyclophilin A, beta-2-microglobulin, RPLP0, Tubulin for samples 7D post LPS and cyclophilin, RPLP0, HPRT for samples 3D post LPS) by the GeNorm software.

### Western blotting

Gastrocnemius muscle from mice sacrificed 72 hours post LPS was processed and analyzed as previously described^48^. In short, muscle was ground to powder using an N_2_-cooled steel mortar. The powder (~20 mg) was lysed in IP-lysis buffer containing protease inhibitors (Complete; Roche Nederland, Woerden, Netherlands), using a rotating blade tissue homogenizer (Polytron homogenizer, Kinematica). Total protein concentration of the supernatant was determined with a BCA protein assay kit (Pierce Biotechnology, #23225, Rockford, IL) according to manufacturer’s instructions. Laemmli buffer was added and samples were denatured by heating at 100 °C for 5 min. Samples were analyzed by western blot. Briefly, 10 μg of protein per lane were separated on a CriterionTM XT Precast 4–12% or 12% Bis-Tris gel (Bio-Rad Laboratories, Veenendaal, Netherlands) and transferred to a nitrocellulose transfer membrane (Bio-Rad Laboratories) by electroblotting. The membrane was stained with Ponceau *S* solution (0.2% Ponceau S in 1% acetic acid; Sigma-Aldrich Chemie) to control for protein loading. The membranes were blocked, washed in TBS-Tween^0.05%^ and incubated overnight at 4 °C with primary antibodies. A list of used antibodies is provided in Supplementary Table S2. All antibodies were diluted 1/1000 in TBS-Tween^0.05^. Blots were then washed and probed with a horseradish peroxidase-conjugated secondary antibody (Vector Laboratories, Burlingame, CA) and visualized with chemiluminescence (Supersignal West Pico or Femto Chemiluminescent Substrate; Pierce Biotechnology) in a LAS-3000 Luminescent Image analyzer (Fujifilm, Tokyo, Japan). Bands were quantified using the Quantity One software (Bio-Rad, version 4.5.0). All data were corrected for protein loading as determined after PonceauS staining.

### Plasma transfer to assess myogenic fusion in C2C12 skeletal muscle cell cultures

Blood was centrifugated (15 min at 2000xg, 4 °C), plasma was collected and aliquoted, and stored in −80 °C.

CRE-C2C12 (Cre-IRES-PuroR^49^) cells plated and cultured as previously described^46^. After 4 days of differentiation, LV-floxed-Luc^50^ myoblasts were added to the differentiated CRE-C2C12 myotubes to assess Cre-dependent activation of luciferase reporter expression^51^. After 6 hours, medium was changed to medium containing 5% mouse plasma and incubated for 3 days. After washing in PBS, cells were lysed in 75 µl lysis buffer and luciferase expression was measured using a luminometer (Berthold).

### Statistical analysis

Data are shown as means ± SE. Comparisons were computed using SPSS version 22.0. For assessment of significance between treatments and phenotypes an independent-samples T-test was used unless stated differently. To determine whether mice responded differently to treatment over time, a repeated measures two-way ANOVA was used. A *p* value < 0.05 was considered statistically significant. Asterisks above a bar refer to a comparison with the respective control (L vs. C and E + L vs. E). An asterisk above a line refers to a comparison between the bars indicated by the line. 0.05 < *p* < 0.1 was considered a trend.

### Data Availability

The datasets generated during and/or analyzed during the current study are available from the corresponding author on reasonable request.

## Electronic supplementary material

Supplementary information
