# Supplementary material for: Altered protein turnover signaling and myogenesis during impaired recovery of inflammation-induced muscle atrophy in emphysematous mice
Source: Sci Rep. 2018 Jul 17;8:10761. doi: 10.1038/s41598-018-28579-4 (PMC6050248; doi:10.1038/s41598-018-28579-4)
Supplement: Supplementary file 1 — Supplementary information [file 41598_2018_28579_MOESM1_ESM.docx]

**Supplementary information for:**

**Altered protein turnover signaling and myogenesis during impaired recovery of inflammation-induced muscle atrophy in emphysematous mice**

Judith JM Ceelen^1^, Annemie MWJ Schols^1^, Anita EM Kneppers^1^, Roger PHA Rosenbrand^1^, Magda M Drożdż^1^, Stefan J van Hoof^2^, Chiel C de Theije^1^, Marco CJM Kelders^1^, Frank Verhaegen^2^, Ramon CJ Langen^1^

Maastricht University Medical Center, Maastricht, the Netherlands.

^1^ Department of Respiratory Medicine. ^2^ Department of Radiation Oncology (MaastRO)

**
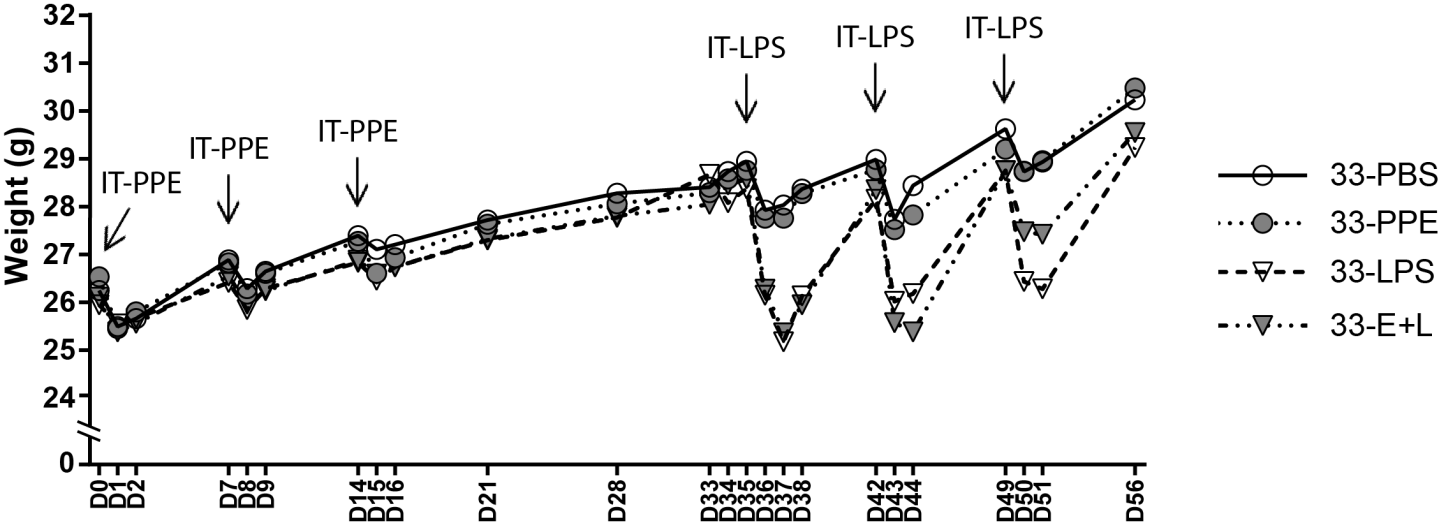
**

**Supplementary figure S1: *No differences in body weight between control and emphysematous mice before and after induction of pulmonary inflammation.*** Mice were intratracheally instilled with elastase to induce emphysema or vc, followed by three weekly instillations with LPS or vc. Body weights of all mice were recorded on the indicated days on the x-axis.

**
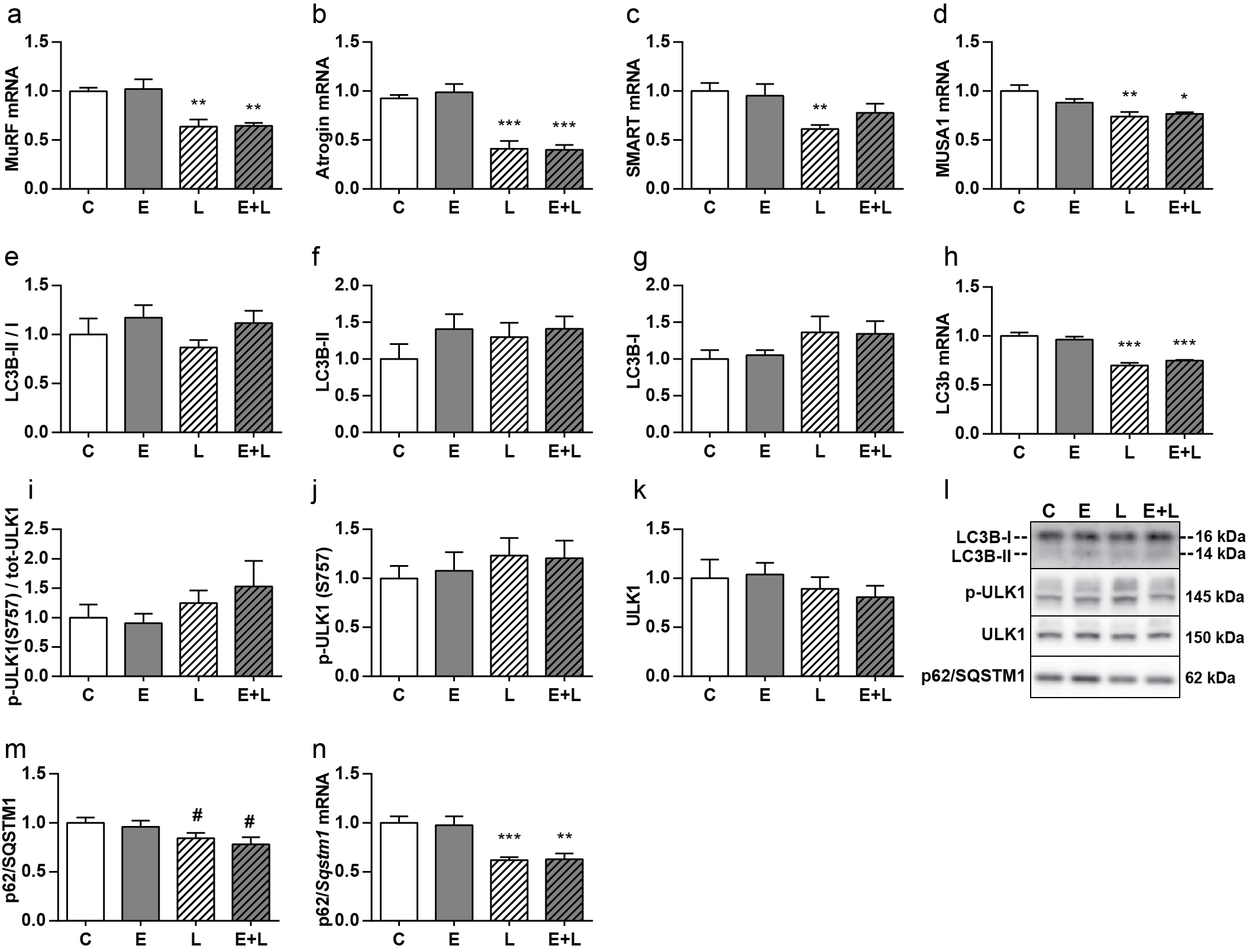
**

**Supplementary figure S2: *No sustained differential regulation of proteolysis signaling between L and E+L mice after recovery from repetitive pulmonary inflammation.*** Mice were intratracheally instilled with elastase to induce emphysema or vc, followed by three weekly instillations with LPS or vc. 7 days after the 3^rd^ LPS gastrocnemius muscle was collected for evaluation of mRNA abundance of **(a)** MuRF1, **(b)** Atrogin-1, **(c)** SMART, **(d)** MUSA1, **(h)** LC3B and **(n)** p62/*Sqstm1*, normalized to GeNorm and expressed as fold change compared to control. Protein levels of **(f)** LC3B-II, **(g)** LC3B-I, **(j)** phosphorylated ULK1 (S757), **(k)** total ULK1 and **(m)** p62/SQSTM1 were assessed in lysates of gastrocnemius muscle tissue with western blot analysis. **(l)** Representative western blots of indicated proteins. Uncropped western blots are shown in Supplementary Fig. S7. Ratio of **(e)** LC3B-II over LC3B-I and **(i)** phosphorylated ULK1 (S757) over total ULK1. n = 6-8 per group. Significant differences were determined using an independent-samples T-test. Symbols above a bar refer to a comparison with the respective control (L vs. C and E+L vs. E). * *p* < 0.05, ** *p* < 0.01, *** *p* < 0.001. # represents a trend.

**
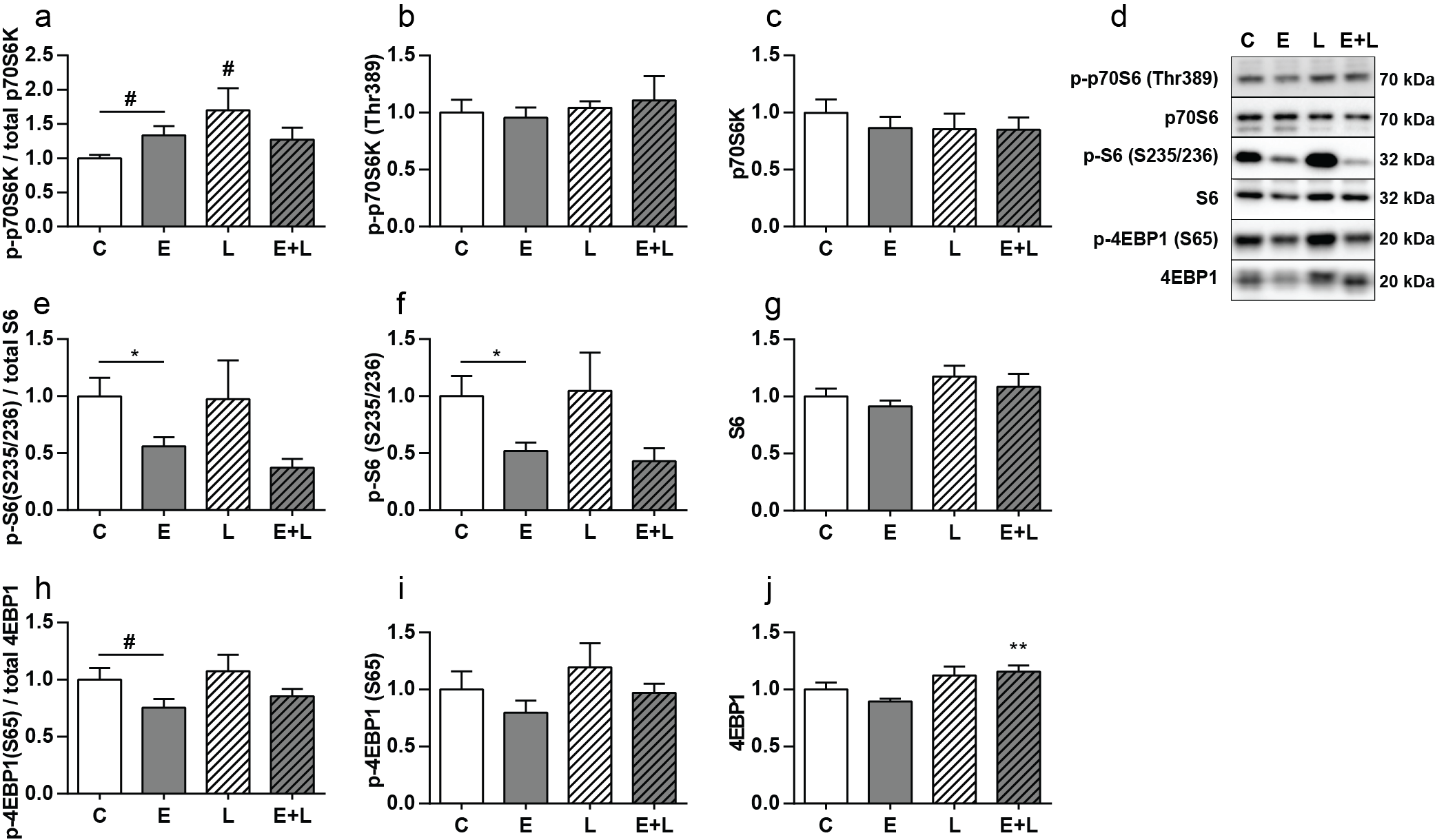
Supplementary figure S3: *Protein synthesis signaling downstream of mTOR in control or emphysematous mice is not affected after recovery from repetitive pulmonary inflammation.*** Mice were intratracheally instilled with elastase to induce emphysema or vc, followed by three weekly instillations with LPS or vc. 7 days after the 3^rd^ LPS gastrocnemius muscle was collected. Protein levels of **(b)** phosphorylated p70S6 (Thr389), **(c)** total p70S6, **(f)** phosphorylated S6 (ser235/236), **(g)** total S6, **(i)** phosphorylated 4EBP1 and **(j)** total 4EBP1 were assessed in lysates of gastrocnemius muscle tissue with western blot analysis. **(d)** Representative western blots of indicated proteins. Uncropped western blots are shown in Supplementary Fig. S8. Ratio of **(a)** phosphorylated p70S6 over total p70S6, **(e)** phosphorylated S6 over total S6 and **(h)** phosphorylated 4EBP1 (ser65) over total 4EBP1. n = 6-8 per group.
Significant differences were determined using an independent-samples T-test. Symbols above a bar refer to a comparison with the respective control (L vs. C and E+L vs. E). A symbol above a line refers to a comparison between the bars indicated by the line. * *p* < 0.05, ** *p* < 0.01, # represents a trend.

**
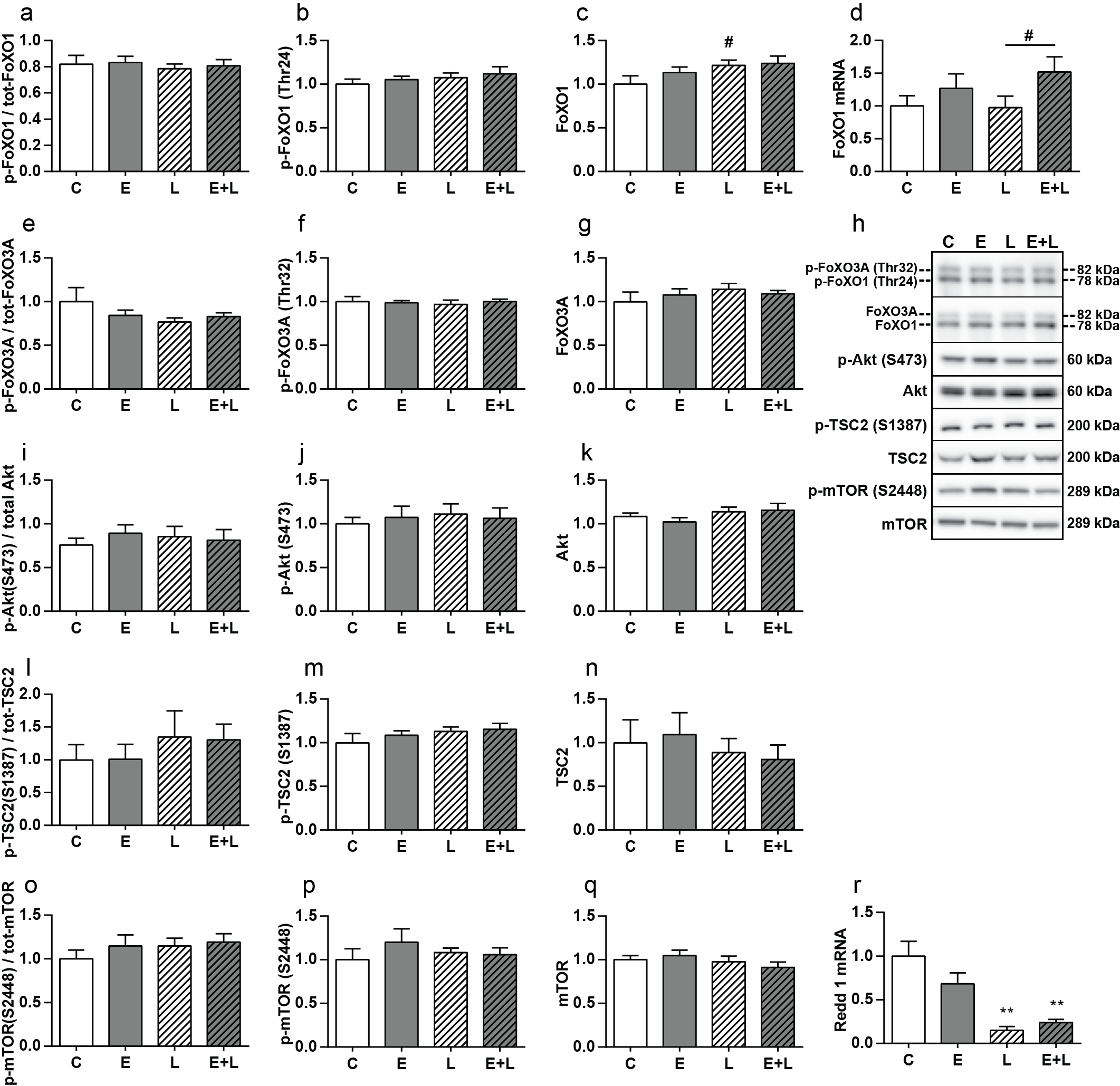
Supplementary figure S4: *No changes in regulatory kinases and transcriptional factors of protein turnover after recovery from repetitive pulmonary inflammation.*** Mice were intratracheally instilled with elastase to induce emphysema or vc, followed by three weekly instillations with LPS or vc. 7 days after the 3^rd^ LPS gastrocnemius muscle was collected. Protein levels of **(b)** phosphorylated FoXO1 (Thr24), **(c)** total FoXO1, **(f)** phosphorylated FoXO3A (Thr32), **(g)** total FoXO3A, **(j)** phosphorylated Akt (S473), **(k)** total Akt, **(m)** phosphorylated TSC2 (S1387), **(n)** total TSC2, **(p)** phosphorylated mTOR (S2448) and **(q)** total mTOR were assessed in lysates of gastrocnemius muscle tissue with western blot analysis. **(h)** Representative western blots of indicated proteins. Uncropped western blots are shown in Supplementary Fig. S9. Ratio of **(a)** phosphorylated FoXO1 over total FoXO1, **(e)** phosphorylated FoXO3A over total FoXO3A, **(i)** phosphorylated Akt over total Akt, **(l)** phosphorylated TSC2 over total TSC2 and **(o)** phosphorylated mTOR over total mTOR. mRNA transcript levels of **(d)** FoXO1 and **(r)** Redd1 were determined, normalized to geNorm, and expressed as fold change compared to control. n = 6-8 per group.
Significant differences were determined using an independent-samples T-test. Symbols above a bar refer to a comparison with the respective control (L vs. C and E+L vs. E). A symbol above a line refers to a comparison between the bars indicated by the line. ** *p* < 0.01, # represents a trend.


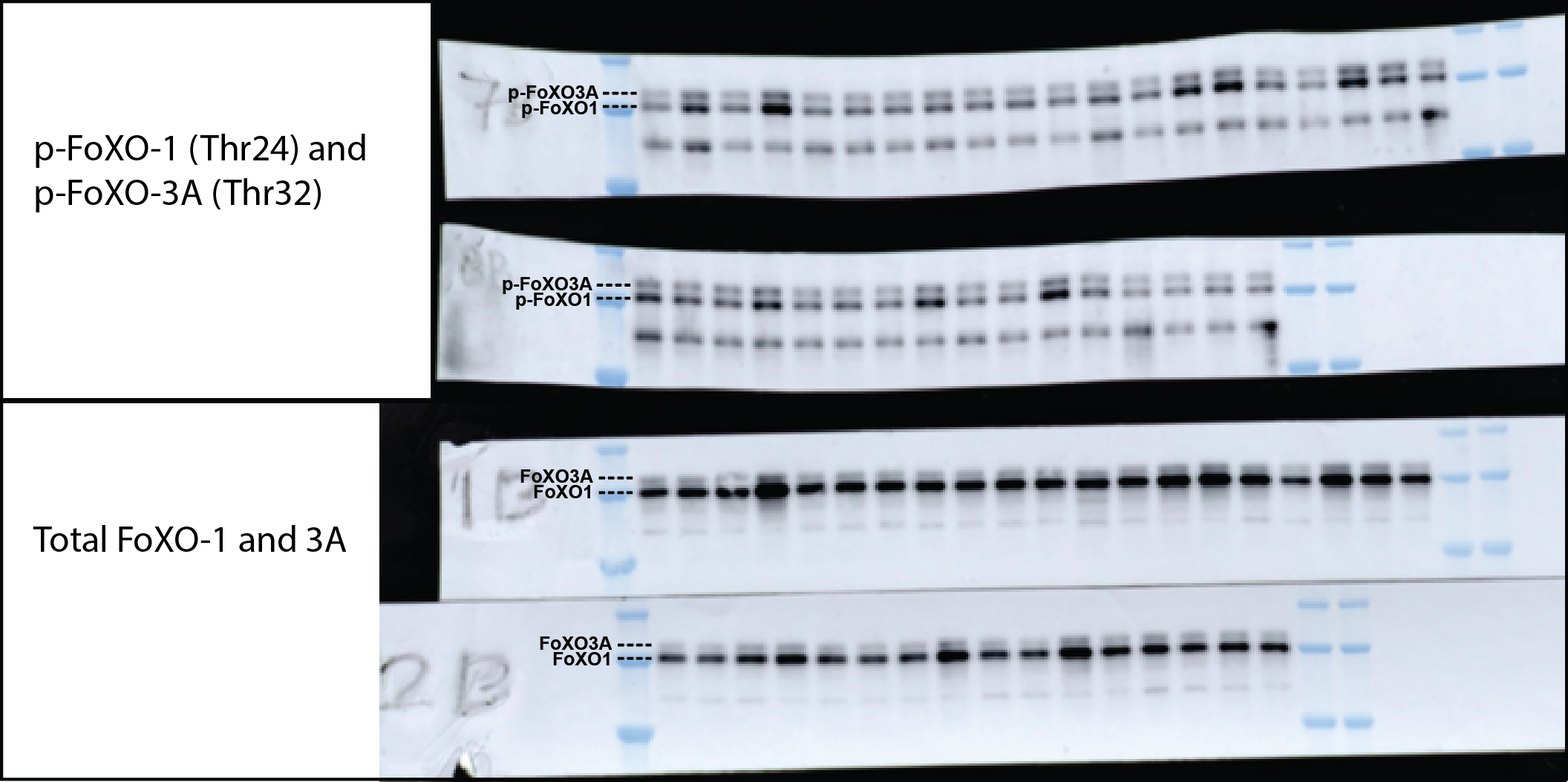


**Supplementary figure S5: *Uncropped western blots from Figure 4.***


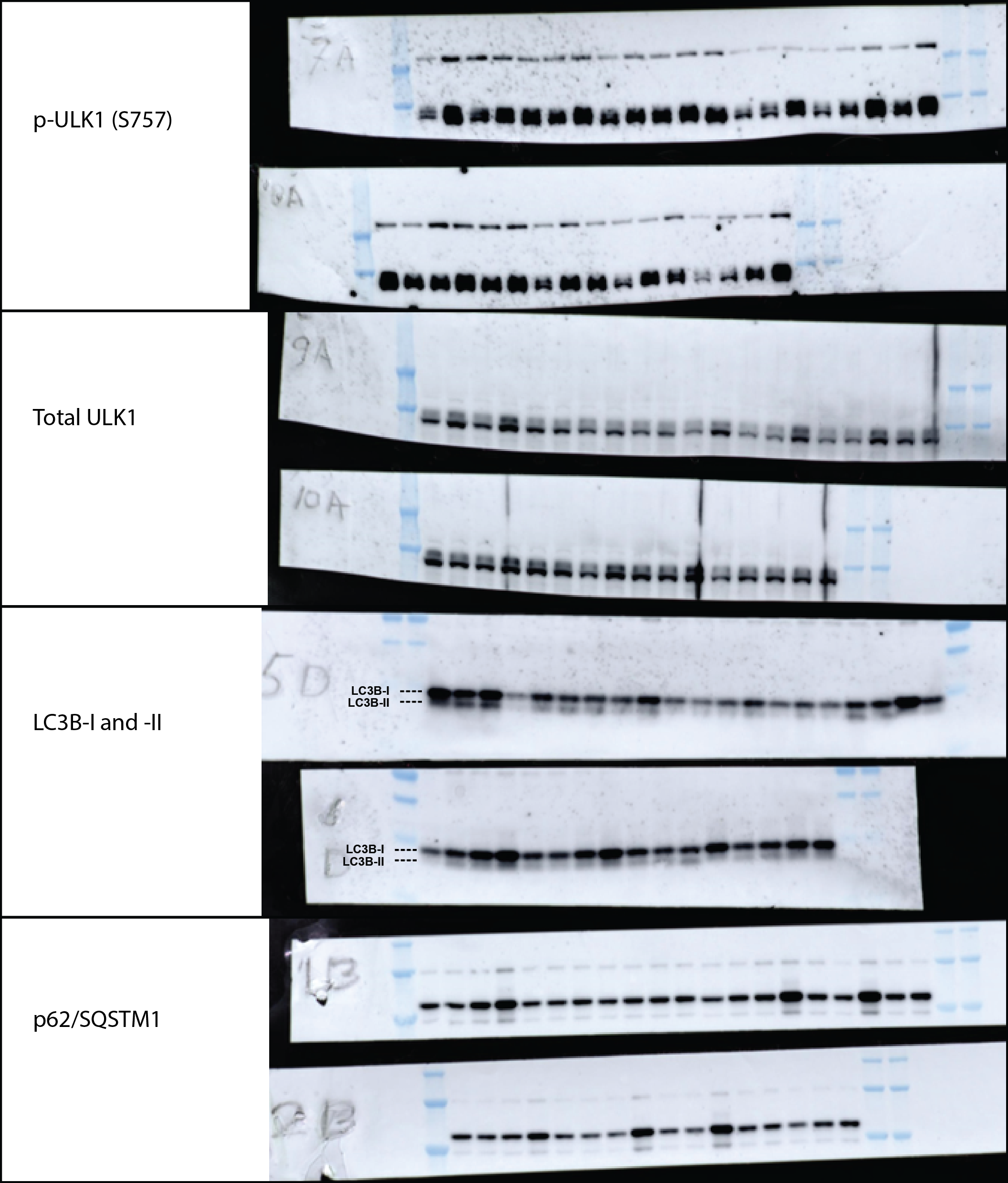


**Supplementary figure S6: *Uncropped western blots from Figure 5.***

**
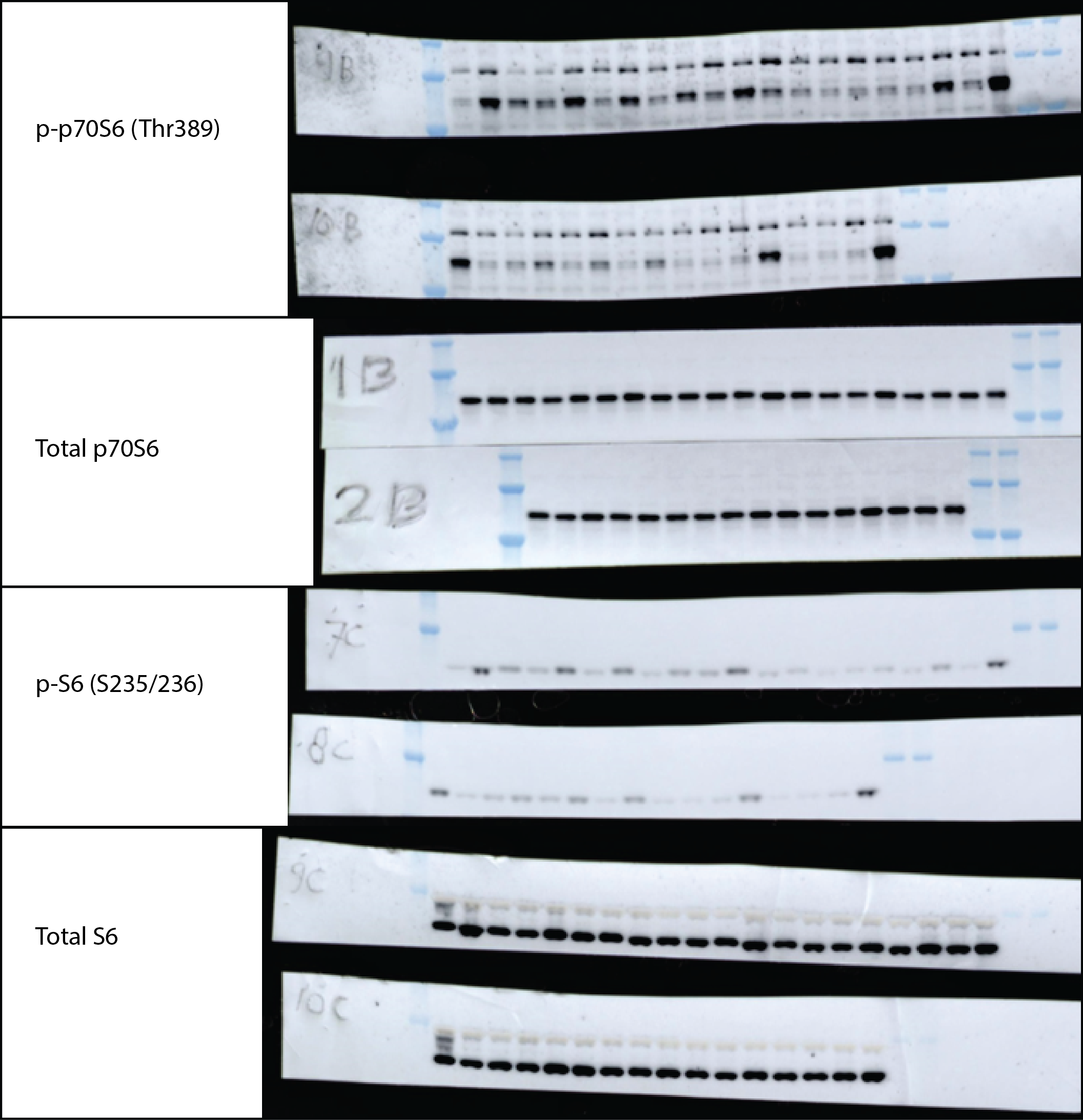
**

**Supplementary figure S7a: *Uncropped western blots from Figure 6.***

**
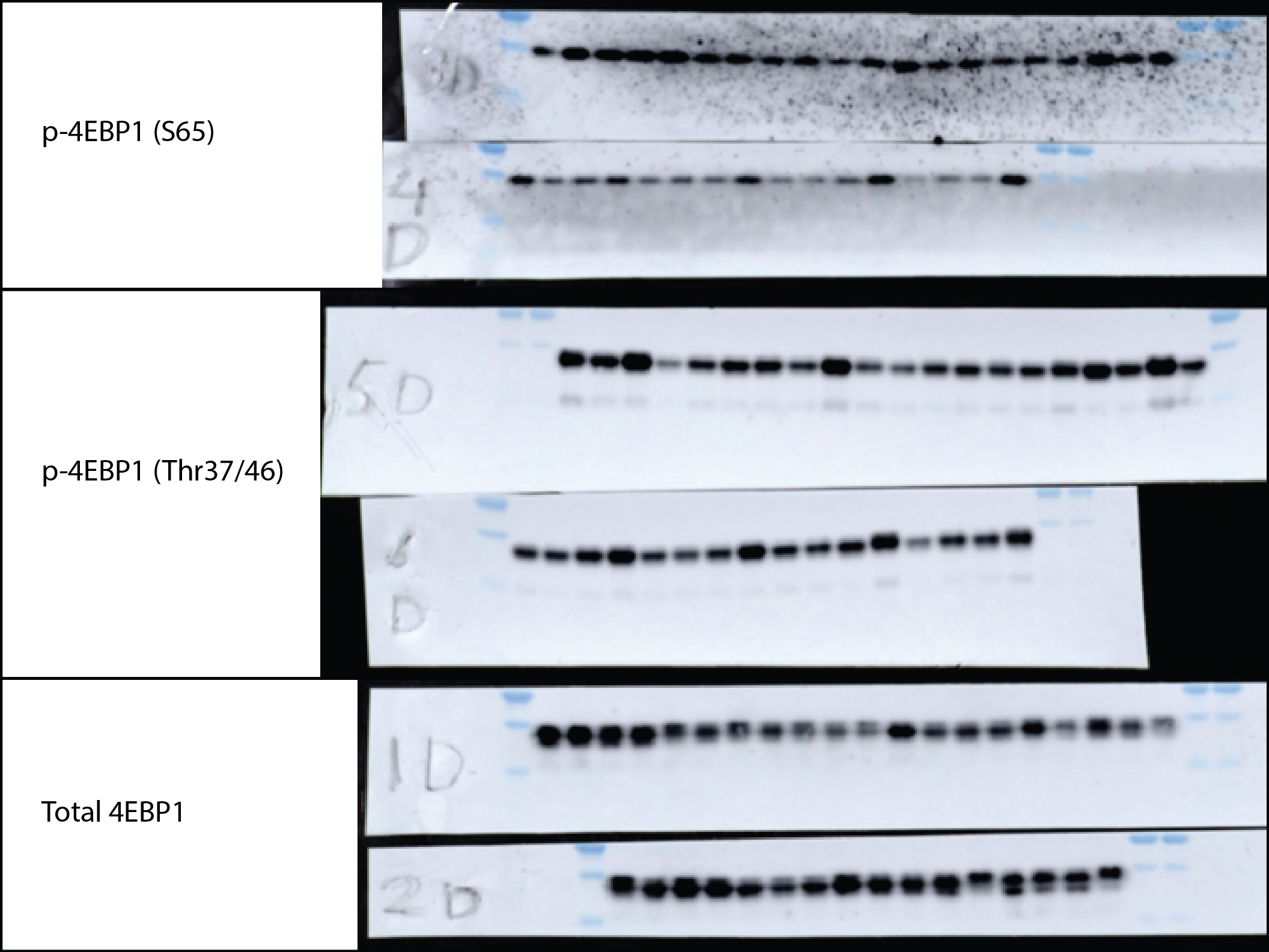
**

**Supplementary figure S7b: *Uncropped western blots from Figure 6.***

**
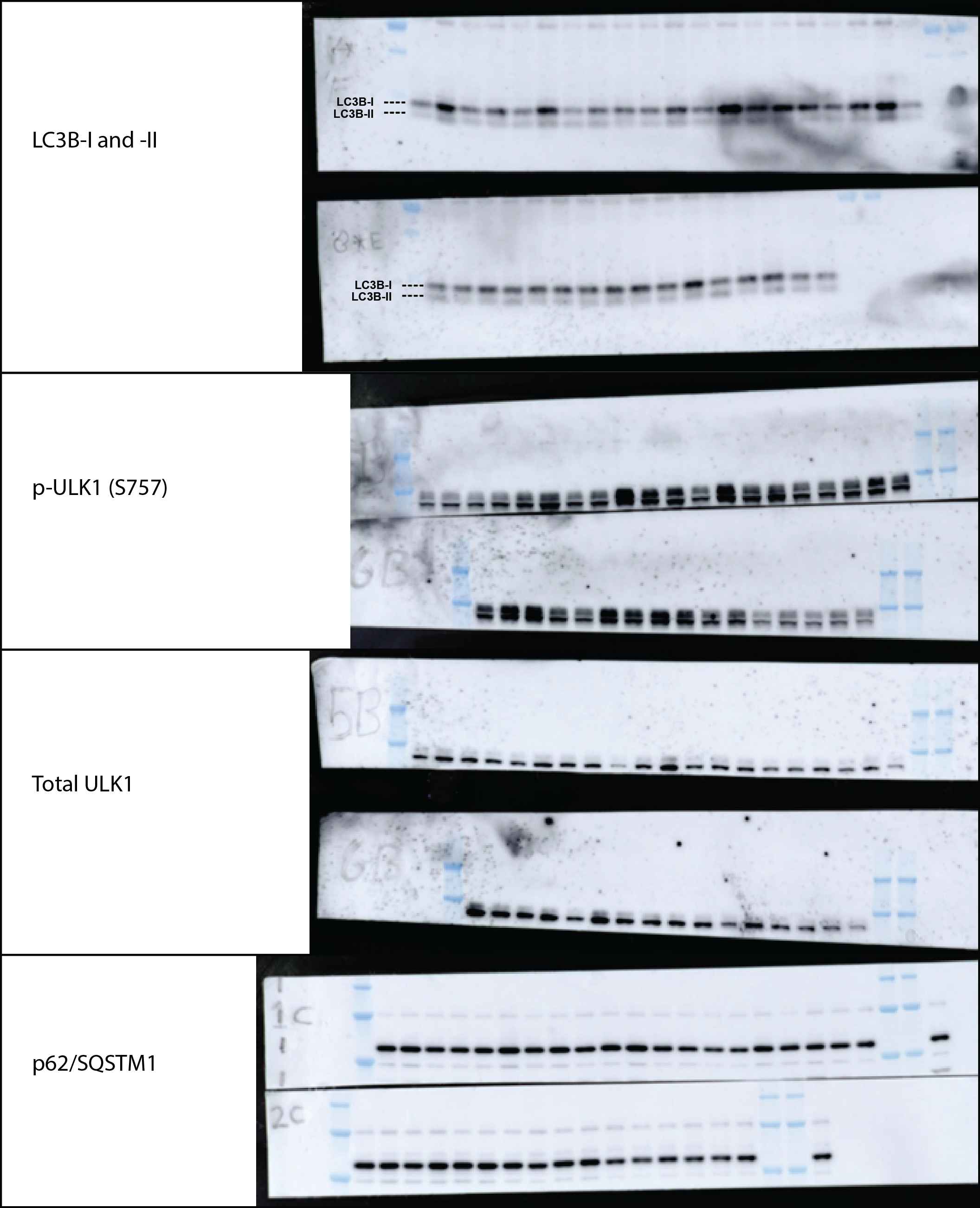
**

**Supplementary figure S8: *Uncropped western blots from Supplementary figure S1.***

**
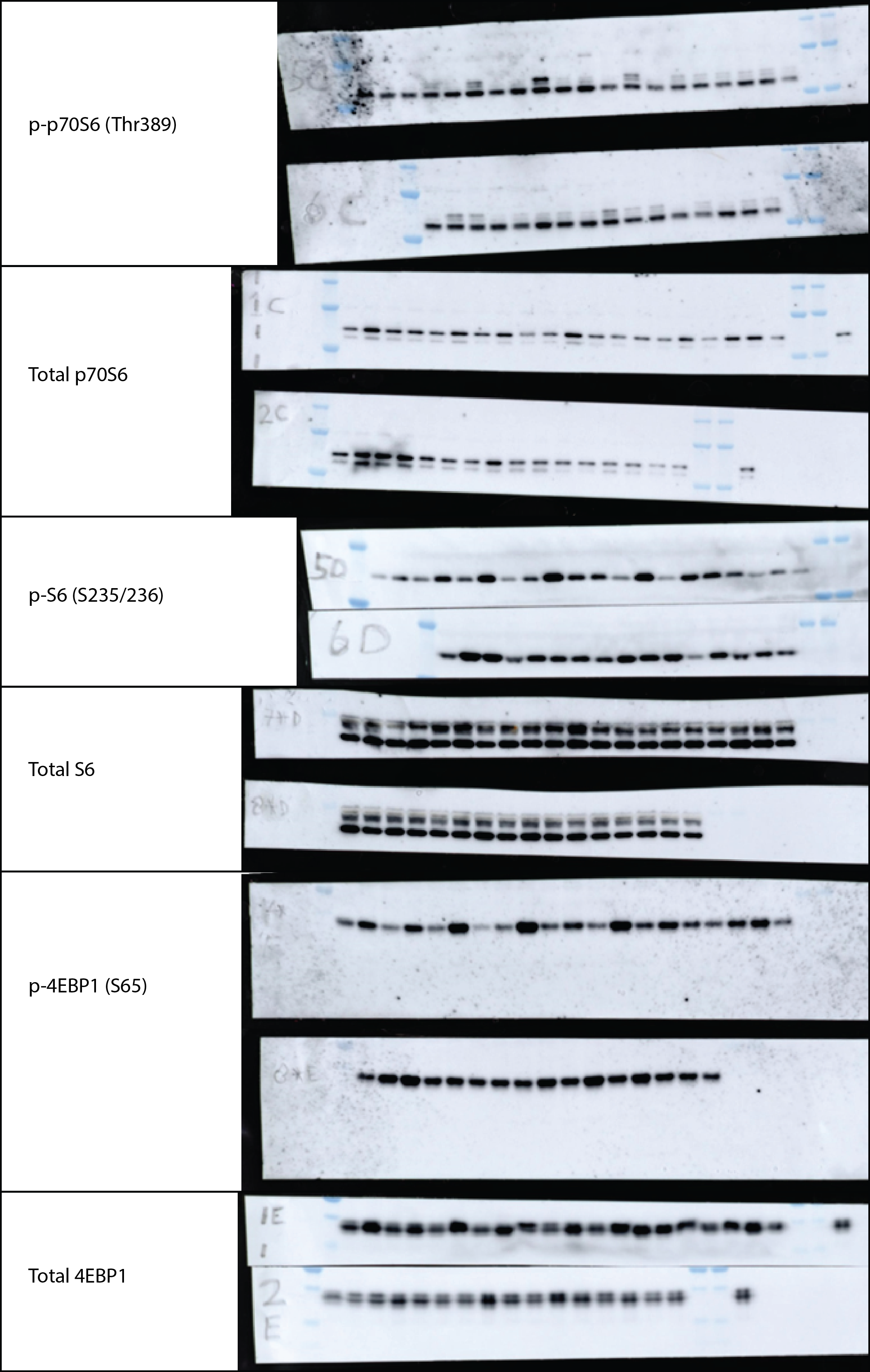
**

**Supplementary figure S9: *Uncropped western blots from Supplementary figure S3.***

**
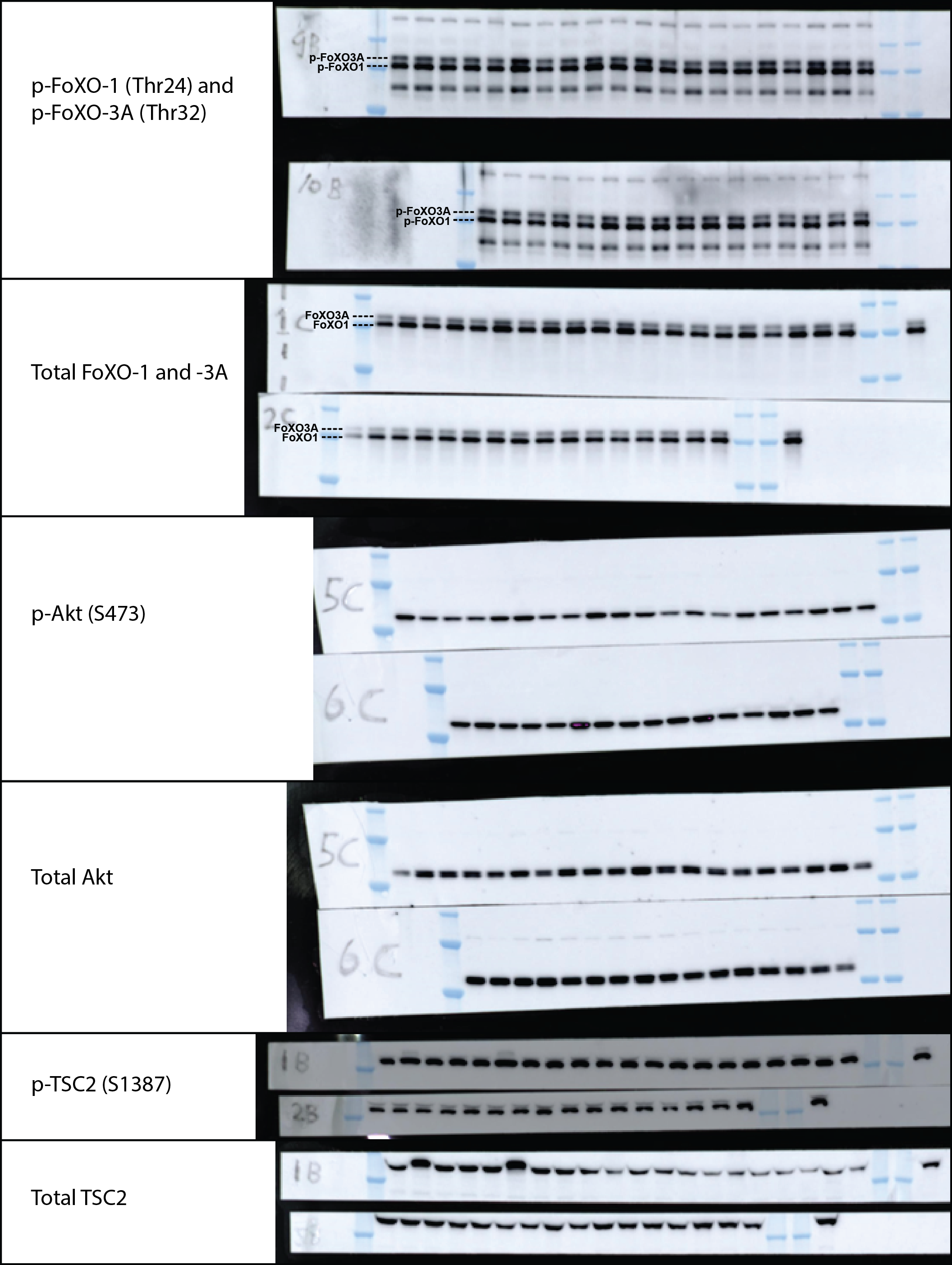
**

**Supplementary figure S10a: *Uncropped western blots from Supplementary figure S4.***

**
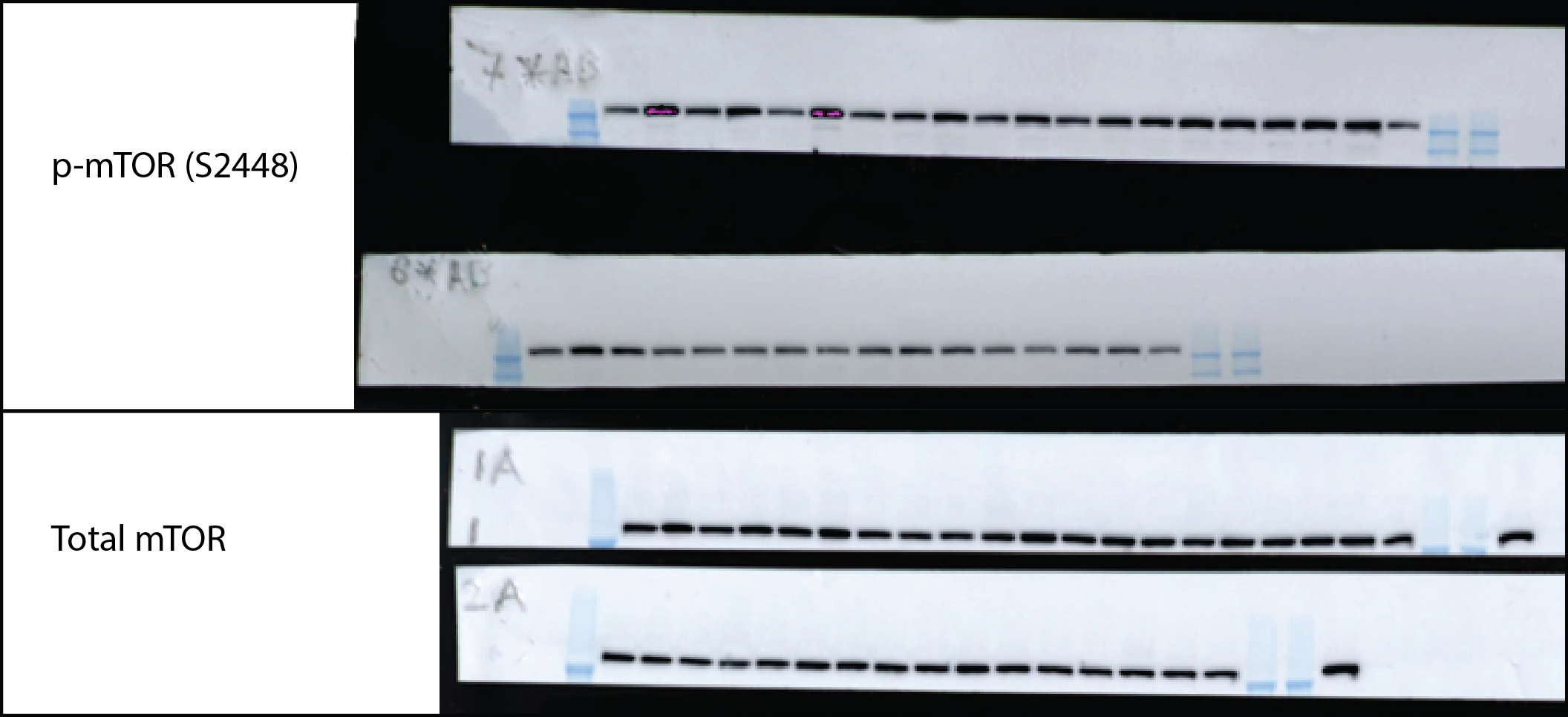
**

**Supplementary figure S10b: *Uncropped western blots from Supplementary figure S4.***

Supplementary table S1: Sequences of primers used for RT-qPCR to assess expression of the indicated genes

| Gene | Forward primer (5’ to 3’) | Reverse primer (5’ to 3’) |
| --- | --- | --- |
| Cyclophilin A | TTCCTCCTTTCACAGAATTATTCCA | CCGCCAGTGCCATTATGG |
| Beta-2-microglobulin | CTTTCTGGTGCTTGTCTCACTGA | GTATGTTCGGCTTCCCATTCTC |
| RPLP0 | GGACCCGAGAAGACCTCCTT | GCACATCACTCAGAATTTCAATGG |
| Tubulin | CGTAGACCTGGAACCCACGGT | TGCCTGTGATGAGCTGCTCA |
| HPRT | TGGATATGCCCTTGACTATAATGAGTAC | AGGACTCCTCGTATTTGCAGATTC |
| KC | TCGTCTTTCATATTGTATGGTCAACACG | TGCCCTACCAACTAGACACAAAATGTC |
| MCP-1 | CCTGCTGTTCACAGTTGCC | ATTGGGATCATCTTGCTGGT |
| CD68 | TGACCTGCTCTCTCTAAGGCTACA | TCACGGTTGCAAGAGAAACATG |
| F480 | CTTTGGCTATGGGCTTCCAGTC | GCAAGGAGGACAGAGTTTATCGTG |
| IκBα | GCTACCCGAGAGCGAGGAT | GCCTCCAAACACACAGTCATCA |
| MuRF1 | CTTCCTCTCAAGTGCCAAGCA | GTGTTCTAAGTCCAGAGTAAAGTAGTCCAT |
| Atrogin-1 | CAGCAGCTGAATAGCATCCAGAT | TCTGCATGATGTTCAGTTGTAAGC |
| SMART | CACAGGGATGTCTGCTACTC | ACACAGTTGTAGCCGTACCTC |
| MUSA1 | GGACGTTTGTGGCAGTTTACTTC | GCAGTACTGAATCGCCATACCTTC |
| FoXO1 | AAGAGCGTGCCCTACTTCAAGGATA | CCATGGACGCAGCTCTTCTC |
| LC3B | GAGCAGCACCCCACCAAGAT | CGTGGTCAGGCACCAGGAA |
| p62/*Sqstm1* | GAATGTGGGGGAGAGTGTGG | TCTTCTGTGCCTGTGCTGGA |
| REDD1 | TCGGCGCTTCACTACTGACC | CCTAACACCCACCCCATTCC |
| Myostatin | GGCCATGATCTTGCTGTAACCT | CGGCAGCACCGGGATT |
| Myomaker | CTGGCCGACTTTGATGAACC | TGCTCTTGTCGGGGTACAGG |
| MyoD | GGCAGAATGGCTACGACACCG | CTTCCCTGGCCTGGACTCGC |
| Myogenin | CCCATGGTGCCCAGTGAA | GCAGATTGTGGGCGTCTGTA |
| MyHC-peri | ACACATCTTGCAGAGGAAGG | TAAACCCAGAGAGGCAAGTG |
| Pax7 | ACCAGTACAGCCAGTATGGCCA | GTGTTCCCCAAGCTTCATACGG |

Supplementary table S2: Antibodies used for western blot

| Antibody | Cat. number | Antibody | Cat. number |
| --- | --- | --- | --- |
| p-FoXO1(Thr24)/FoXO3A(Thr32) | #9464 | p-S6 (S235/236) | #4856 |
| FoXO1 | #2880 | S6 | #2217 |
| FoXO3A | #2497 | p-p70S6 (Thr389) | #9205 |
| LC3B | #2775 | p70S6 | #9202 |
| p-ULK1 (S757) | #6888 | p-mTOR (S2448) | #2971 |
| ULK1 | #8054 | mTOR | #2983 |
| p62/SQSTM1 | #5114 | p-Akt (S473) | #9271 |
| p-4EBP1 (S65) | #9451 | Akt | #9272 |
| p-4EBP1 (Thr37/46) | #9459 | p-TSC2 (S1387) | #3617 |
| 4EBP1 | #9452 | TSC2 | #4308 |
